# Modeled Benefit of Individual Cancer Signal Origin Prediction for Multi-Cancer Early Detection

**DOI:** 10.1158/2767-9764.CRC-24-0351

**Published:** 2025-05-19

**Authors:** Eric A. Klein, Timothy R. Church, Christina A. Clarke, Earl Hubbell

**Affiliations:** 1 GRAIL, Inc., Menlo Park, California.; 2 Division of Environmental Health Sciences, University of Minnesota School of Public Health, Minneapolis, Minnesota.

## Abstract

Multi-cancer early detection (MCED) tests may detect a broad spectrum of cancer types, including uncommon types that lack recommended screening. After a cancer signal is detected by an MCED test, some diagnostic process must definitively confirm the patient’s cancer status. A commercially available blood-based MCED test detects a cancer signal and then predicts an anatomic location, a cancer signal origin (CSO), to guide the diagnostic process. We extended a preexisting model for MCED cancer screening, adding predicted CSO categories and a simple model of the diagnostic chain. We then predicted outcomes of the diagnostic chain for each predicted CSO and in populations with differing clinical risk factors. Typical positive predictive values were>40%, and using a minimal sufficient level of positive predictive value (>7%), (i) diagnostic workup based on any CSO was generally warranted, and (ii) continued workup for cancers in locations other than the CSO was justifiable. The benefit of prediction-directed workups was also observed via estimated clinical utility metrics, such as lives saved per diagnostic test, and remained applicable in populations with varying cancer risk, such as lung cancer prediction-directed workups in never-smokers. CSO predictions may enable most true-positive cases to be resolved by short and efficient diagnostic processes. The model predicted a large enough conditional benefit to warrant diagnostic workup based on any CSO prediction from an MCED test, assuming late-stage reduction by MCED leads to mortality reduction, which remains to be demonstrated.

**Significance:**

MCED tests may detect a signal from many cancers. Predicting an anatomic location from which the cancer signal may originate allows effective, usual diagnostic workup. In this study, we show that these predictions are beneficial to physicians choosing a diagnostic path, even for uncommon cancer types and among populations with differing cancer risks.

## Introduction

Cancer is the second leading cause of death in the United States (1, 2). Multi-cancer early detection (MCED) tests that detect cell-free DNA shed by invasive cancer cells offer the potential to decrease overall cancer mortality by finding cancers not currently targeted by existing screening programs and by shifting diagnosis to earlier stages (2–6). This potential reduction in late-stage diagnoses and, ultimately, cancer mortality may be realized if the MCED test can also accurately predict the cancer signal origin (CSO) to direct focused and efficient diagnostic evaluations.

One commercially available blood-based MCED test (Galleri; GRAIL, Inc.) uses a targeted methylation assay and machine learning classifier to measure the extent and location of methylation in cell-free DNA to infer a cancer-specific methylation pattern across a diverse set of invasive cancer types (5–7). When a cancer signal is detected, a second classifier provides a most probable, molecular-based CSO prediction to assist in guiding diagnostic workup, which had an overall accuracy of 89% (6) in the third Circulating Cell-Free Genome Atlas substudy (CCGA3; NCT02889978), a large case–control study. In that same study, aggregate test specificity and sensitivity of the MCED test for cancer signal detection were 99.5% and 51.5%, respectively.

Because it is not feasible to evaluate all possible screening strategies and resulting diagnostic chains in clinical trials, modeling plays a key role in assessing the utility of new screening technologies. Previous work highlighted the potential of an MCED test to shift cancer detection from late to early stages, ultimately reducing overall cancer mortality in the general U.S. population ages 50 to 79 years by up to 26% (8). This shift depends on timely and effective diagnosis following screen detection. Although current safety studies show empirical results on the diagnostic chain, they are small (9). Detailed modeling of the diagnostic chain following a cancer signal–detected result is limited in the literature, leading to questions about appropriate implementation of MCED tests and recommendations for diagnostic procedures.

Previous studies have modeled features of implementing an MCED test, with a range of strengths and limitations. One model duplicates the single false positive (FP) rate across cancer types (10, 11). By assuming that each cancer type is targeted by a separate test, positive predictive value (PPV) calculations for individual cancers may be underestimated, and the FP rates accumulate across cancer types. Another model treats an MCED test as a collection of single-cancer tests that screen for a preselected set of target cancers instead of one that detects a shared cancer signal associated with multiple cancer types (see Supplementary Materials) (12). This approach neglects the potential effects on and from additional cancer incidence from cancers not preselected and terminates diagnostic chains after a single guided step; the remaining individuals are still at significant risk of cancer. A third model assumes the use of whole-body imaging as part of the diagnostic chain to eliminate the need for a CSO prediction (4, 13). This assumption does not resemble standard practice in healthcare systems (14–16), such as the National Health Service 2-week wait or “urgent suspected cancer” pathways (17, 18). In addition, the FDA recently advised against this imaging approach, noting that a tissue-of-origin component (e.g., CSO prediction) guiding targeted diagnostic workup is important to minimize the risks associated with diagnostic tests, underscoring the importance of modeling the effects of CSO prediction (19).

**Figure 1:**
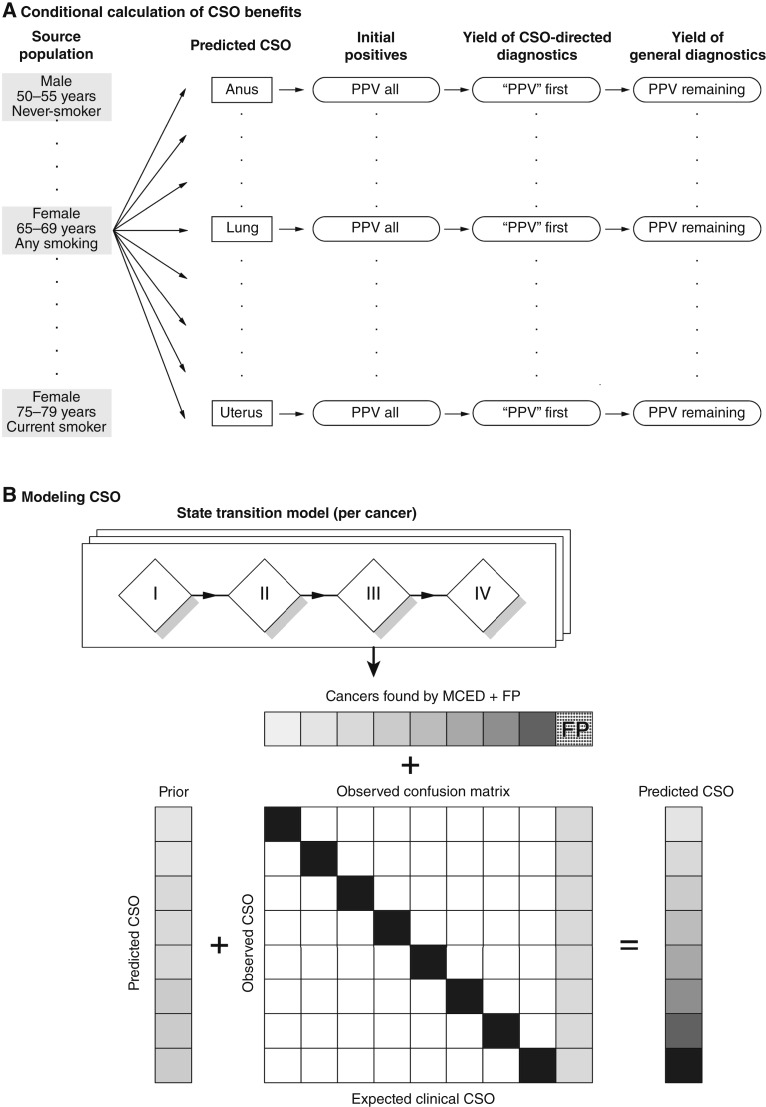
**A,** Schematic representation of the model. For each source population, we compute the incidence of each cancer type and predict what cancers will be found and the predicted CSO, including FPs. For each successive step in the pathway, we compute the fraction of initial positives that are true cancer cases (PPV all), the fraction likely to be found by a CSO-directed diagnostic (PPV first), and the remaining risk of cancer in individuals for which a given CSO has been ruled out (PPV remaining). **B,** Modeling the assignment of CSO predictions to cancer signal–detected results. A previously published state-transition model generates estimates of cancer types and stages found by MCED, as well as the FP rate. For each cancer type and associated clinical CSO, the observed confusion matrix obtained from the validation study provides an estimate of the probability of observing a CSO given the clinical expected CSO (columns). As this confusion matrix has combinations that were not observed (but are possible in practice), we supply a prior based on the expected classifier behavior to fill in the probability of these rare events. Similarly for FPs, we estimate the probability of observing each CSO for a FP. Combining these potential observed CSOs across the rows, we obtain the final observed incidence of each CSO, and can separate out the contributions from cancers expected to have this CSO, cross-talk from all other cancers, and FPs.

In this study, we addressed these issues by modeling a multi-step diagnostic chain, modeling invasive cancer incidence across all cancer types, and incorporating the conditional benefit of following a CSO-directed diagnostic chain (Fig. 1A). We abstracted the diagnostic process by dividing the diagnostic chain into individual steps, each of which may contain several actions taken to resolve a case. We note two types of diagnostic steps: those directed toward ruling in or out a particular site of cancer, corresponding roughly to 2-week wait pathways, and those directed toward ruling in or out nonlocalized (non–site-specific) cancer risk. In general, site-directed steps are standard for most medical care; non–site-specific diagnostic steps may consist of whole-body imaging or retesting using a blood-based MCED test to confirm the accuracy of the initial result.

We built upon an existing model that estimates rates of detection and associated stage at diagnosis (8) and extended it to include a CSO prediction for individuals in whom a cancer signal is detected, adding both cross-talk between cancer cases (incorrect CSO predictions) and CSO predictions for FPs (Fig. 1B). This improved model allows us to quantitatively assess the utility of providing a CSO prediction and estimate variations in clinical utility based on underlying clinical factors in a general U.S. population, such as sex, smoking history, and age.

## Materials and Methods

### CSO prediction model overview

We modeled the potential actions and outcomes conditional on each CSO prediction that may be provided alongside a cancer signal–detected result after an MCED test in a general screening age population (Fig. 1A). CSO predictions were added to an existing population model of cancers intercepted and resulting lives saved after cancer signal detection from an MCED test (8). Briefly, CSO predictions were modeled per potential CSOs returned using the confusion matrix developed from CCGA3 (6), augmented with prior probabilities of classifier error. This augmented model returned a CSO prediction for all positives: true positives (TP) with the correct CSO prediction, TPs with an incorrect CSO prediction, and FPs, who are individuals with no cancer present. It is important to account for TPs with incorrect CSO predictions, as well as CSO predictions associated with FPs, because the diagnostic chain can be chosen based only on information provided, not the underlying true cancer state of an individual. We then modeled a diagnostic chain to resolve each case.

### Cancer incidence data for the U.S. population within the age range of 40 to 84

We obtained cancer registry data from the NCI’s Surveillance, Epidemiology, and End Results (SEER; RRID: SCR_006902) program (diagnosis years 2006–2015), including all individuals ages 40 to 84 years diagnosed with any invasive cancer in 18 geographic regions of the United States, including follow-up survival data through 2020. Cancer types were divided into 25 cancer classes (6, 8), including “other” cancers not associated with any single class. These classes were further subdivided to separate out neuroendocrine tumors, which have unique methylation patterns separate from their host organs and an associated CSO. SEER incidence was also stratified by sex and age by 5 year categories and adjusted for smoking status using risks from a previously published study (20).

### Performance of an MCED test

Data from a case–control study of a currently commercially available MCED test were used to characterize both the detection rates of cancer types by stage as well as the accuracy of the CSO prediction (6). We further used a FP rate as reported in that study. We note that the MCED test has a single FP rate for cancer signal detection, such that the number of individuals without cancer who enter any diagnostic process is fixed and does not change with the number of CSO categories into which they are stratified. Some individuals may have more complex diagnostic processes than others, but the number of individuals who receive diagnostic processes (with or without cancer) is the same as the number of individuals with positive signals.

### Detection and stage at diagnosis

We estimated the detection rate and the stage of diagnosis, as previously described (8). This state-transition model (Supplementary Fig. S1), in which cancer evolves from earlier stages to later stages, allows an MCED test to find a detectable cancer at a stage before clinical diagnosis (Supplementary Methods). The probability of finding a cancer at a given stage depends on the fraction of cancers shedding DNA at that stage (sensitivity), as well as the time spent in each stage (dwell time).

### CSO frequency calculations

We modeled CSO predictions for cancer signal–detected results separately for true cancer cases (TPs) and FPs (Fig. 1B). Given a clinical CSO for an individual with cancer, the model produces a conditional probability of predicting each CSO for that individual. For each clinical CSO in CCGA3, there is a set of CSO predictions. However, the finite validation set cannot exclude low-probability events, and we therefore augment the observed data with a reference prior to avoid zero probabilities for unobserved events. This prior augmentation assigns each CSO a predicted probability based on the frequency of clinical CSOs among the individuals who received a cancer signal–detected result, matching the probability distribution in the CSO classifier training data (Supplementary Data). For FPs, which are sparser due to the high specificity of the test, the reference prior is similarly combined with the few observed predictions for FPs in CCGA3. Using these predicted probabilities, for each cancer signal–detected result in the basic model, whether TP or FP, we assign a rate for each possible CSO prediction.

### Model of diagnostic process

We modeled a simple diagnostic chain of individual steps, each of which may contain several actions taken to resolve a case. We noted two types of diagnostic steps: CSO-directed cancer-specific evaluations, and non–site-specific evaluations. The model first applies a diagnostic test driven by a CSO prediction for cancers of that type. After this initial test, the model computes the remaining individuals with that CSO prediction, estimating the potential for applying a general diagnostic test (e.g., whole-body imaging). The model treats these two diagnostic tests as having 100% sensitivity for cancer of that type and 0% sensitivity for cancers of other types (see “Discussion”).

### Computation of the PPV

For each step in the diagnostic chain, the model can define a PPV (the fraction of TPs given the current group of individuals with a given set of results). It then computes the number of TPs of any cancer type within each predicted CSO, the number of TPs matching the expected CSO within each predicted CSO (those whom the specific diagnostic test will find), and the number of TPs remaining for the last step in the diagnostic chain (individuals for whom that cancer has been ruled out). Individuals with cancer are TPs for cancer, regardless of the predicted CSO used to stratify them into a diagnostic chain. FPs remain in the pool until ruled out by a general test.

### Computation of diagnostic steps needed to save a life

Lives saved were computed for each step in the diagnostic chain using the simple predictor of differences in 5-year survival. Briefly, within each CSO prediction, we estimate the number of lives saved by finding cancers, both for CSO-directed and non–site-specific (post-CSO prediction) diagnostic evaluations. We then count the number of diagnostic steps of each type needed to save a life within each CSO prediction.

### Stochastic variability and sensitivity analysis

Briefly, draws from the posterior distribution of the parameter estimates from external studies are fed into the state-transition model to produce variability in output (see Supplementary Materials) corresponding to uncertainty in input parameters. We do not explicitly model cases of decreased sensitivity or increased FPs separately, as they are covered within the range of variation. For sensitivity analysis of dwell time, variation in dwell time (decreasing time available for detection of cancers) using the fast aggressive scenario previously described (8) was used and compared with the results of this analysis.

For mortality analyses, overall survival (OS) by age was used to account for competing risks. In a separate sensitivity analysis, an increased HR was postulated in cancer-specific survival for cancers shedding circulating tumor DNA (ctDNA) vs cancers not shedding ctDNA in a way that maintained the average observed survival in SEER.

### Strategic analysis

Alternate strategies that involved terminating workup after initial CSO-directed workup (forgoing any undirected workup) and that separately forewent any CSO-directed workup were analyzed for the relative number of tests and types of tests used, as well as residual risks at termination of workup.

### Data availability

The data generated in this study are available upon request from the corresponding author. The code and data used in this study are available at https://github.com/grailbio-publications/Klein_CSO_Benefit.

## Results

We estimated the conditional benefit of each CSO prediction currently available as part of an MCED test using performance metrics (PPV, number of diagnostic tests, and lives saved) in specific subpopulations of interest within the general population ages 40 to 84 years and stratified the results by sex, smoking status, and detailed age, shown here within the usual screening range of 50 to 79 years (55–59, 65–69, or 75–79 years). We used a PPV of 7% as a minimal reasonable threshold for clinicians considering diagnostic workup decisions after cancer signal detection (21). Additionally, we used the modeled benchmark of 240 diagnostic mammograms needed to save one life across the population (22) to reflect typical levels of justified diagnostic effort.

### Modeled PPV stratified by CSO

**Figure 2:**
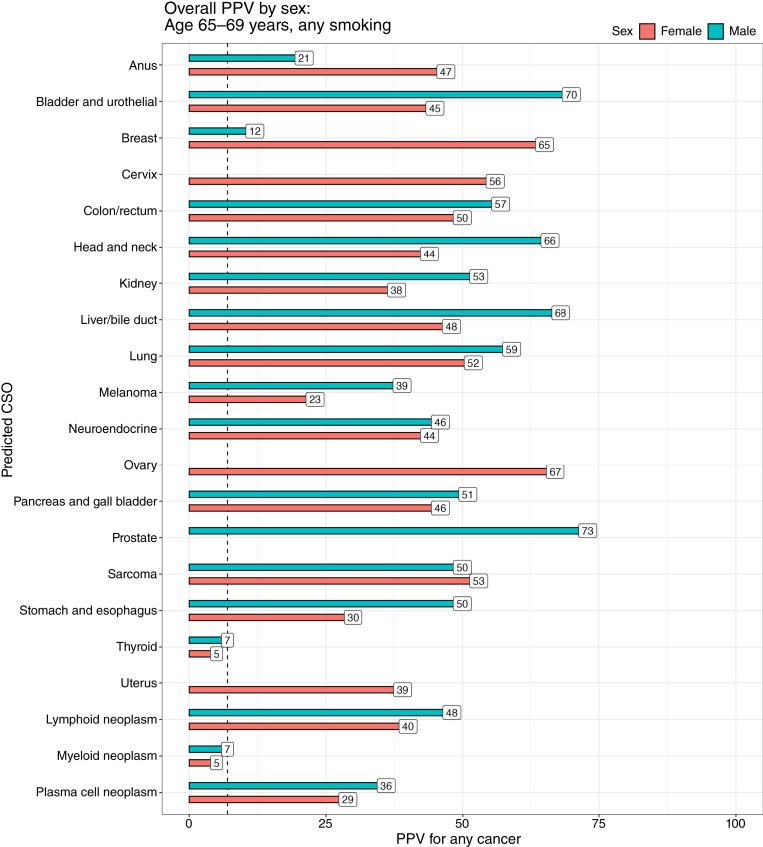
Overall PPVs modeled for each CSO prediction by sex. This reflects the fact that every predicted CSO has sufficient cancers in general to justify a future diagnostic workup, even if the occurrence of that CSO is infrequent. Here, we see that despite breast cancer being uncommon in men, there is still enough risk of cancer in a predicted breast CSO to justify investigation of cancer. The dashed line represents a threshold PPV of 7%, typically justifying workup.

**Figure 3:**
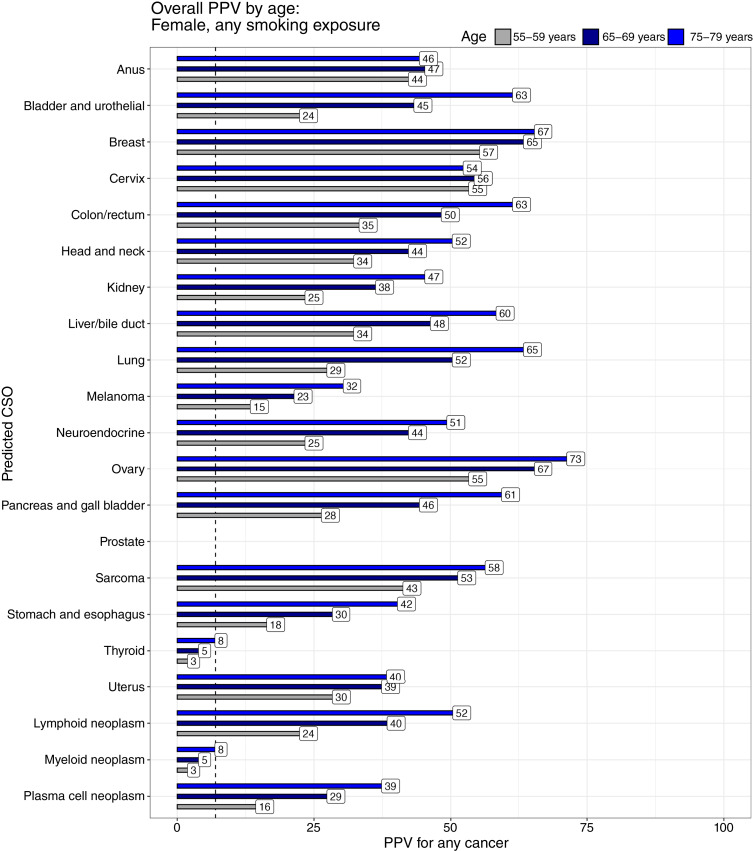
Overall PPVs modeled for each CSO prediction by age. Cancer incidence increases with age, and in this model, FPs are constant across age groups, so the PPV increases with age. Importantly, despite minor changes in relative incidence of cancer types with age, the PPV still indicates a noticeable risk of cancer within each age range. The dashed line represents a threshold PPV of 7%, typically justifying workup.

**Figure 4:**
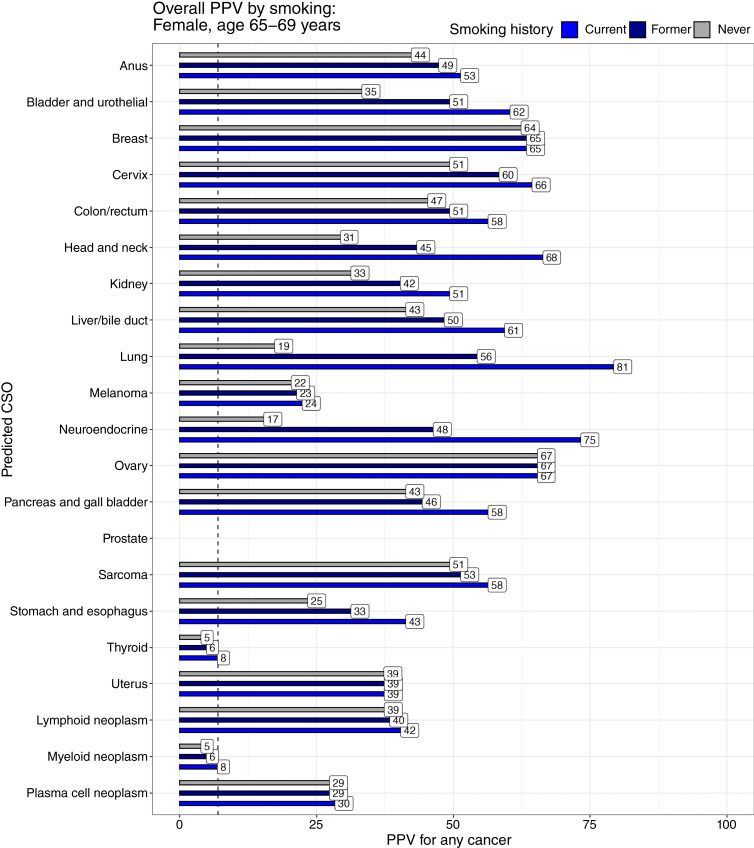
Overall PPVs modeled for each CSO prediction by smoking status. Smoking alters the incidence of multiple cancers, most strikingly lung and neuroendocrine (primarily small cell lung cancer). Even never-smokers have a significant risk of cancer when a lung CSO is predicted. In Supplementary Data, this includes a significant quantity of lung cancer. The dashed line represents a threshold PPV of 7%, typically justifying workup.

For a 65- to 69-year-old female, the modeled PPV for any cancer for each predicted CSO had median (range) 44.5% (myeloid 5%, ovary 67%; Fig. 2). Similarly, the modeled PPV for a 65- to 69-year-old male had median 50% (myeloid 7%, prostate 73%). Even in such a male, a CSO prediction of breast had a PPV of 10% (Supplementary Table S1). By age, the modeled PPV had median 28.5% (3%–57%) for a 55- to 59-year-old female and median 52% (8%–73%) for a 75- to 79-year-old female (Fig. 3). Stratified by smoking status, the PPV for a 65- to 69-year-old female (medians: never-smoker 37%, current smoker 58%) varied from a low of 5% (never-smoker, myeloid) to 81% (current smoker, lung). Strikingly, lung—the predicted CSO most affected by smoking—had a PPV of 19% in never-smokers, rising to 81% in current smokers (Fig. 4).

**Figure 5:**
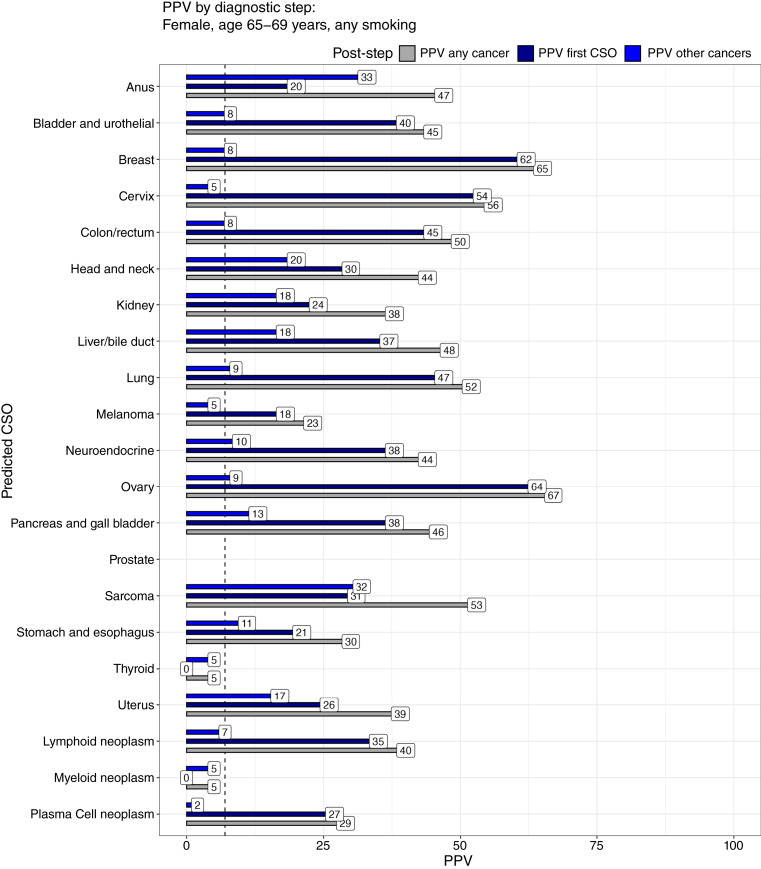
All PPVs in the modeled diagnostic chain for a 65- to 69-year-old female with any smoking status. Here, we break out the fraction of cancer in individuals with each CSO in the starting pool (PPV any cancer), the fraction of the specific cancer in individuals with each specific CSO (PPV first CSO), and, finally, in individuals who do not have that specific cancer found, how many other cancers are expected (PPV other cancers). For almost every cancer type, there is sufficient predictive power to justify diagnostic workup for that particular cancer (except those with no evidence of detectable ctDNA in studies, such as thyroid, or no successful CSO predictions, such as myeloid). Similarly, it is worthwhile to investigate the remaining individuals for other cancers, as previously reported (32). Whereas there are some patterns in the cross-talk between cancers and predicted CSOs, we do not specifically model a second-best targeted diagnostic investigation. The dashed line represents a threshold PPV of 7%, typically justifying workup.

Examining specific steps in the diagnostic chain using a 65- to 69-year-old-female as an example (Fig. 5), we see that the PPV (fractional yield) for the specific diagnostic test at a predicted CSO ranged from 18% to 64% (melanoma, ovary; median: 36%), excluding cancer types for which there was no evidence in CCGA3 of detection (thyroid) or no successful CSO predictions in CCGA3 (myeloid). The remaining cancer risk (PPV after that cancer is ruled out) ranged from 2% to 33% (plasma cell neoplasm, anus; median: 9.5%).

### Diagnostic tests and lives saved

**Figure 6:**
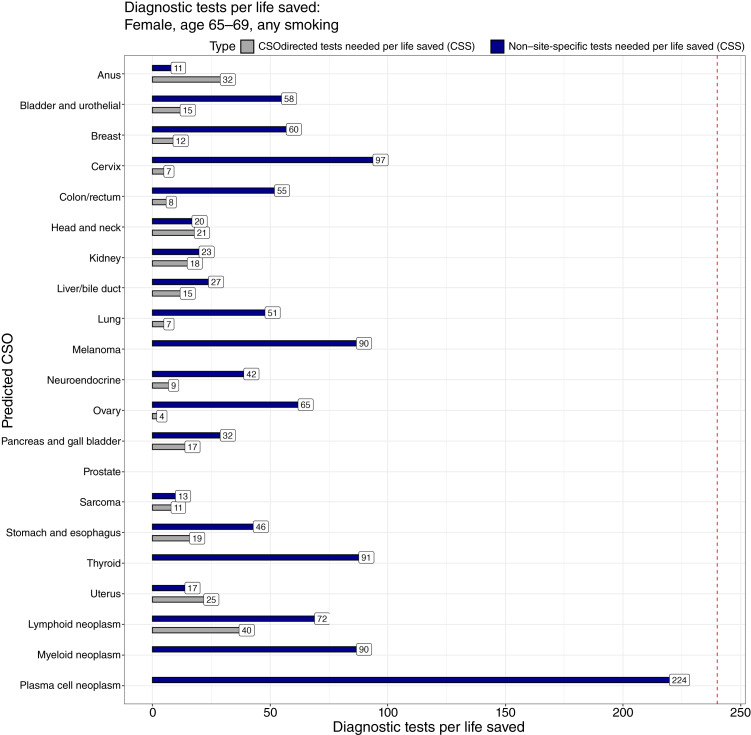
Number of 65- to 69-year-old females with any smoking history who need to undergo a diagnostic test to save one life for each CSO. After a cancer signal–detected result, as diagnostic tests are done at each step of the diagnostic chain, we can estimate an approximate clinical tradeoff in terms of diagnostic tests needed to save a life. Across nearly all predicted CSOs, this tradeoff is much better than that for diagnostic mammography. For some predicted CSOs, the particular cancer is either unstaged, has no specific survival data by stage reliably recorded, or has no modeled stage shift. In those cases, the bar is missing in the plot as the number of tests to save one life could not be estimated. The dashed line represents a modeled benchmark of 240 diagnostic mammograms needed to save one life.

In the example population of 65- to 69-year-old females, anywhere from 4 to 40 (ovary, lymphoid; median: 15) specific diagnostic steps across the population may be needed to save one life, excluding four cancer CSOs not modelable for this statistic due to small numbers of detections in CCGA3 (myeloid, thyroid, plasma, and melanoma; Fig. 6; of note, the reference line for mammography is at 240 diagnostic tests to save a life). For the vast majority of CSOs, it may take ≤20 specific diagnostic tests across the population to save a single life (12/16 modeled CSOs for the first diagnostic step; see Methods for a description of modeled CSOs). If the specific diagnostic test directed by a CSO is inconclusive, the number of general diagnostic tests across the population that may be needed to save a life ranged from 11 to 97 (anus, cervix; median: 44), with the majority below 60 (12/16 total predictable CSOs for females). Full performance data tables for incidence rounds of screening and prevalence rounds of screening can be found in Supplementary Materials: Supplementary Tables S1 and S2, respectively. Data supporting Figs. 5 and 6 are presented in Supplementary Table S1.

### Stochastic variability and sensitivity analysis

Briefly, in models incorporating uncertainty in input sensitivity data and FP rate estimates, the majority of output variation is driven by uncertainty in the FP rate (Supplementary Figs. S2–S6, corresponding to Figs. 2–6; Supplementary Figs. S7 and S8); however, the PPV of most cases as well as lives saved per diagnostic test still remain in a clinically actionable zone. Reducing dwell times for sensitivity analysis has the effect of increasing the number of interval cancers (decreasing episode sensitivity), which reduces both the PPV and lives saved; however, again, both remain in clinically actionable zones for the majority of cases (Supplementary Figs. S9–S12).

Altering mortality tradeoffs by either including competing risks by age (OS, Supplementary Figs. S13 and S14) or increasing the HR for survival in those detected by MCED (Supplementary Figs. S15 and S16) do not directly affect the PPV but alter the lives-saved calculation. Again, because of an extremely large margin for most individual CSO–population combinations, the majority of cases remain in a clinically actionable zone.

### Strategic analysis

Alternate strategies of only using CSO-directed workups and then stopping preserve the majority of lives saved and minimize the use of expensive undirected workups; however, the residual risk of cancer remains in the clinically actionable (unacceptably large) range after such minimization. Performing only undirected workups and ignoring CSO information leads to an increased number of undirected tests, mostly occurring in TPs whose diagnosis would have been confirmed by less burdensome directed tests. Ratios between test numbers are shown in Supplementary Figs. S17 to S20.

## Discussion

In this study, we extend previous work and generate a detailed computational model not only for detection with an MCED test but also the diagnostic chain that follows a positive test with a CSO prediction. Within this framework, we modeled the PPV and lives saved upon diagnostic workup conditional on each predicted CSO. Judging by existing benchmarks for diagnostic decision-making given a risk-of-cancer indication, this model predicted potential conditional benefit large enough to warrant diagnostic workup directed by a CSO prediction of cancer signal–detected results from an available MCED test despite orders of magnitude difference in the expected cases receiving each CSO prediction (Supplementary Table S1).

Whereas the lowest median PPV for following a particular CSO for choosing a diagnostic path illustrated is 28.5% (55- to 59-year-old female), it is important to assess the worst-case behavior for individual CSOs. Across almost all predicted CSOs within most modeled populations, the PPV for any cancer (or for a cancer associated with a specific clinical CSO matching the predicted CSO) was greater than 7% (Supplementary Data), which is a reasonable threshold for clinicians considering diagnostic workup decisions after cancer signal detection (21). This PPV threshold is comparable with two representative benchmarks: first, the likelihood that cancer is detected in individuals with Li–Fraumeni syndrome (those who are at genetic risk of cancer due to a *TP53* gene alteration) during recommended whole-body MRI (22, 23); and second, the PPV of Guy’s Rapid Diagnostic Clinic in England dedicated to the diagnostic workup for individuals with increased suspicion of cancer (24).

Although the PPV indicates the fraction of individuals among those testing positive on screening for the condition tested (cancer) to be referred for diagnosis, and hence the potential for diagnostic procedures to succeed, it does not measure the clinical utility of those procedures. A simple metric for clinical utility is estimated lives saved per diagnostic (not screening) procedure. Modeling of the mammography screening and diagnostic chain shows that one life is saved for every 240 diagnostic mammograms (25). Using a surrogate measure of lives saved in this model (differences in predicted 5-year survival), the number of diagnostic tests needed to save a life was considerably lower, both for an initial diagnostic workup directed by a CSO prediction and for a later round of non–site-specific diagnostic evaluations (Fig. 6). This suggests that—across nearly all populations and CSO predictions—CSO-directed workups may have clinical utility and that continuing workups are justified, even after the first workup is negative for a particular cancer.

Unlike single-cancer tests, for which FP rates accumulate across multiple cancer types (26), an MCED test has a single FP rate that limits the number of individuals experiencing any diagnostic process. Although some individuals require additional workups to finally locate or exclude an existing cancer, in practice, these workups have been observed to terminate in a relatively short amount of time without undue harm (9). The potential for overdiagnosis is also limited, first by the high specificity, which reduces the number of individuals in which any indolent lesions may be found by chance, and second by the observed biological properties of ctDNA-detectable cancers. Biophysical models suggest that slow-growing tumors and indolent small lesions do not shed detectable amounts of ctDNA (27), and prognostic indications suggest that ctDNA-detectable cancers are not indolent (28–31).

A few limitations are important to consider. For simplicity, calculations assumed 100% sensitivity for diagnostic testing subsequent to a cancer signal detected MCED test result, whereas most diagnostic tests do have some error rate (32). Adapting the model to imperfect diagnostic tests does not change the trend of the results. As previously modeled, CSO-directed diagnostic workup effectively diagnoses cancer cases, but often, after an initial negative diagnostic workup, there is a residual risk of cancer, warranting further investigation at the original site, as well as other sites (32).

Additionally, calculation of lives saved did not account for potential mortality from unnecessary tests or diagnostic pursuit of incidental findings, though any mortality from these diagnostic tests is expected to be small compared with the projected benefit of early detection. Screening is generally not recommended for individuals with severe comorbidities that either greatly shorten lifespan or make invasive procedures intolerable. Furthermore, we do not specifically account for competing risks in the main analysis that may limit lifespan and hence limit the benefit for cancer detection, as individuals in the modeled age range generally have more than 10 years of life remaining. To account for competing risks, changes in OS are examined in Supplementary Materials. Whereas this reduces the lives-saved metric, numbers of diagnostic tests to save a life continue to be far below the average for mammography in the screening age population.

Modeled mortality benefits from cancer screening due to reduction in late-stage cancer incidence are inferred from stage-specific survival in this study, as clinical trials with a cancer-specific mortality endpoint have not been completed yet as they require large populations and long times to observe; however, a large-scale clinical trial of MCED screening utilizing reduction in late-stage cancer incidence as the primary endpoint is expected to read out shortly (33). We rely here on the strong correlation between reduction in late-stage cancer incidence and cancer-specific mortality outcomes observed across previous trials of cancer screening (34). Other endpoints, such as reduction in late-stage cancer incidence, are being proposed as short-term intermediate endpoints for clinical studies; however, care must be taken that such intermediate endpoints are chosen and modeled to properly reflect mortality (35–37). Accepting these limitations, in this study, we have modeled both reduction in late-stage cancer incidence and mortality changes to allow plausible scenarios to be explored.

The modeled accuracy of each CSO prediction was taken from the clinical validation study for the currently available MCED test (6); however, because that study had so few FP results, the distribution or rate of FPs across CSO predictions may be different in particular populations. This will affect the conditional distribution of the PPV for each predicted CSO (38). The small number of FPs leads to high potential variability in the ultimate observed FP rate from 0.3% to 0.8%, which affects the PPV and number of tests required (Supplementary Materials). There is further uncertainty in the sensitivity values due to finite sampling of cancer cases in the clinical validation study (6). We show the potential variability in the results due to both of these factors in Supplementary Materials. Additionally, this model used historic SEER incidence data, modified by appropriate risk factors, to estimate cancer incidence and outcomes in each population.

In this work, harms of screening were simplified to unnecessary diagnostic steps, but actual harms are more complex and vary by specific diagnostic tests used and the site of investigation. Generally, site-specific investigations are justified by an estimated 3% to 5% risk of cancer due to symptoms or other causes, such as in the National Health Service 2-week wait or “urgent suspected cancer” pathways (17, 18). This characterizes the risk of harm from site-directed diagnostic pathways that is balanced by the projected benefits of early detection.

Additionally, the MCED test was assumed to have similar performance between subpopulations with different distributions of risk factors. We do not specifically model any tests tuned for a particular subpopulation. Real-world data are needed to confirm the modeled results.

Consistent with the previously stated preferences of the FDA, our modeling suggests that CSO predictions have the potential to save time and expense over undirected workup for general cancer risk (39, 40), reducing expensive and sometimes invasive tests and the risk of high radiation exposure, as well as (41) incidental findings, and more easily fitting into standard physician workflows. However, this benefit depends on the relative difficulty and expense of non–CSO-directed testing routines to CSO-directed workups (Supplementary Materials). Indeed, if the difficulty of non–site-directed tests is high enough, only CSO-directed tests may be used, with some loss of potential lives saved (Supplementary Materials). The remaining need to manage additional non–site-specific cancer risk after negative CSO-directed workups may be addressed by other means of ruling out FP signals other than whole-body imaging, such as retesting to confirm an initial cancer signal detected after a negative diagnostic workup, which has shown good results in clinical practice (42). Research into methods to improve diagnostic resolution of FP status, similar to nodule management protocols in low-dose CT (43, 44), should be a priority to maximize the clinical utility of MCED tests.

In summary, we improve models of MCED test performance by explicitly modeling the effect of a given CSO prediction on the resulting diagnostic chain. Importantly, this reflects the published performance of a commercial MCED test, although the model is generally applicable to any MCED test returning a CSO prediction (6). Site-directed workups are commonly performed in patients who present with organ system–specific symptoms suspicious for cancer; as such, adoption of CSO prediction-directed diagnostic evaluations fits with current clinical practice.

## Supplementary Material

Supplementary Materials Supplementary Methods, Data, and References

Supplementary Figure 1 State transition diagram for the interception model, expanded to show the five possible trajectories of cancer detectability by stage, and the potential detection by MCED or by usual care

Supplementary Figure 2 Overall PPVs modeled for each cancer signal origin prediction by sex, accounting for uncertainty in sensitivity, specificity, and cancer signal origin assignment

Supplementary Figure 3 Overall PPVs modeled for each cancer signal origin prediction by age, accounting for uncertainty in sensitivity, specificity, and cancer signal origin assignment

Supplementary Figure 4 Overall PPVs modeled for each cancer signal origin prediction by smoking status, accounting for uncertainty in sensitivity, specificity, and cancer signal origin assignment

Supplementary Figure 5 All PPVs in the modeled diagnostic chain for 65- to 69-year-old females with any smoking status, accounting for uncertainty in sensitivity, specificity, and cancer signal origin assignment

Supplementary Figure 6 Number of 65- to 69-year-old females with any smoking history who need to undergo a diagnostic test to save one life for each cancer signal origin, accounting for uncertainty in sensitivity, specificity, and cancer signal origin assignment

Supplementary Figure 7 Relationship between PPV and diagnostic tests to save a life for CSO-directed workups, shown stratified by cancer signal origin, colored by sex

Supplementary Figure 8 Relationship between PPV and diagnostic tests to save a life for post-CSO-directed workups, shown stratified by cancer signal origin, colored by sex

Supplementary Figure 9 Overall PPV for any cancer, stratified by cancer signal origin with comparison across dwell time scenarios

Supplementary Figure 11 PPV plotted against diagnostic tests per lives saved across age ranges and sexes, stratified by dwell time scenario

Supplementary Figure 10 Diagnostic tests per life saved for all cancer signal origins, comparison across dwell time scenarios

Supplementary Figure 12 PPV plotted against diagnostic tests per lives saved, comparison between dwell time scenarios for post-CSO-directed workups

Supplementary Figure 13 Diagnostic tests per lives saved for CSO-directed workups, age bands covering 50-80 years, incidence as default for SEER (“any” smoking status as smoking status is unknown in SEER)

Supplementary Figure 14 Diagnostic tests per lives saved for post-CSO-directed workups, age bands covering 50-80 years, incidence as default for SEER

Supplementary Figure 15 Diagnostic tests per life-saved with increased relative hazard for cfDNA detectable cancers, CSO-directed workups

Supplementary Figure 16 Diagnostic tests per life-saved with increased relative hazard for cfDNA detectable cancers, post-CSO-directed tests

Supplementary Figure 17 Breakdown of total numbers of tests by type under either a strategy using CSO-directed workups followed by post-CSO non-CSO-directed workups

Supplementary Figure 18 Effective ratios of “expense” to favor CSO-directed workups, stratified by age and smoking exposure

Supplementary Figure 19 Reduction in lives saved from a strategy only using CSO-directed workups and stopping any further workup, stratified by age, sex, and smoking exposure

Supplementary Figure 20 Residual risk of cancer in individuals after CSO-directed testing, stratified by age, sex, and smoking exposure

Supplementary Table 1 Extrapolated performance numbers

Supplementary Table 2 Extrapolated prevalence performance numbers

Supplementary Materials Input Parameters

## References

[1] Siegel RL , Giaquinto AN, Jemal A. Cancer statistics, 2024. CA Cancer J Clin 2024;74:12–49. 38230766 10.3322/caac.21820

[2] Cristiano S , Leal A, Phallen J, Fiksel J, Adleff V, Bruhm DC, . Genome-wide cell-free DNA fragmentation in patients with cancer. Nature 2019;570:385–9. 31142840 10.1038/s41586-019-1272-6 PMC6774252

[3] Cohen JD , Li L, Wang Y, Thoburn C, Afsari B, Danilova L, . Detection and localization of surgically resectable cancers with a multi-analyte blood test. Science 2018;359:926–30. 29348365 10.1126/science.aar3247 PMC6080308

[4] Lennon AM , Buchanan AH, Kinde I, Warren A, Honushefsky A, Cohain AT, . Feasibility of blood testing combined with PET-CT to screen for cancer and guide intervention. Science 2020 369:eabb9601. 32345712 10.1126/science.abb9601 PMC7509949

[5] Liu MC , Oxnard GR, Klein EA, Swanton C, Seiden MV; CCGA Consortium. Sensitive and specific multi-cancer detection and localization using methylation signatures in cell-free DNA. Ann Oncol 2020;31:745–59. 33506766 10.1016/j.annonc.2020.02.011 PMC8274402

[6] Klein EA , Richards D, Cohn A, Tummala M, Lapham R, Cosgrove D, . Clinical validation of a targeted methylation-based multi-cancer early detection test using an independent validation set. Ann Oncol 2021;32:1167–77. 34176681 10.1016/j.annonc.2021.05.806

[7] Jamshidi A , Liu MC, Klein EA, Venn O, Hubbell E, Beausang JF, . Evaluation of cell-free DNA approaches for multi-cancer early detection. Cancer Cell 2022;40:1537–49.e12. 36400018 10.1016/j.ccell.2022.10.022

[8] Hubbell E , Clarke CA, Aravanis AM, Berg CD. Modeled reductions in late-stage cancer with a multi-cancer early detection test. Cancer Epidemiol Biomarkers Prev 2021;30:460–8. 33328254 10.1158/1055-9965.EPI-20-1134

[9] Schrag D , Beer TM, McDonnell CH, Nadauld L, Dilaveri CA, Reid R, . Blood-based tests for multicancer early detection (PATHFINDER): a prospective cohort study. The Lancet 2023;402:1251–60. 10.1016/S0140-6736(23)01700-2 PMC11027492 37805216

[10] Fiala C , Diamandis EP. A multi-cancer detection test: focus on the positive predictive value. Ann Oncol 2020;31:1267–8. 32741675 10.1016/j.annonc.2020.05.028

[11] Liu MC , Oxnard GR, Klein EA, Swanton C, Seiden M. Response to W.C. Taylor, and C. Fiala and E.P. Diamandis, Ann Oncol 2020;31:1268–70. 32569726 10.1016/j.annonc.2020.06.008

[12] Jiao B , Gulati R, Katki HA, Castle PE, Etzioni R. A quantitative framework to study potential benefits and harms of multi-cancer early detection testing. Cancer Epidemiol Biomark Prev 2022;31:38–44. 10.1158/1055-9965.EPI-21-0380 PMC8755582 34548329

[13] Kisiel JB , Ebbert JO, Taylor WR, Marinac CR, Choudhry OA, Rego SP, . Shifting the cancer screening paradigm: developing a multi-biomarker class approach to multi-cancer early detection testing. Life 2024;14:925. 39202669 10.3390/life14080925 PMC11355654

[14] Damhus CS , Siersma V, Dalton SO, Brodersen J. Non-specific symptoms and signs of cancer: different organisations of a cancer patient pathway in Denmark. Scand J Prim Health Care 2021;39:23–30. 33629891 10.1080/02813432.2021.1880094 PMC7971193

[15] Elliss-Brookes L , McPhail S, Ives A, Greenslade M, Shelton J, Hiom S, . Routes to diagnosis for cancer – determining the patient journey using multiple routine data sets. Br J Cancer 2012;107:1220–6. 22996611 10.1038/bjc.2012.408 PMC3494426

[16] Koo MM , Hamilton W, Walter FM, Rubin GP, Lyratzopoulos G. Symptom signatures and diagnostic timeliness in cancer patients: a review of current evidence. Neoplasia 2017;20:165–74. 29253839 10.1016/j.neo.2017.11.005 PMC5735300

[17] National Health Service . NHS cancer plan [Internet]; 2000. [cited 2024 May 30]. Available from: https://webarchive.nationalarchives.gov.uk/ukgwa/20130107105354/http://www.dh.gov.uk/prod_consum_dh/groups/dh_digitalassets/@dh/@en/documents/digitalasset/dh_4014513.pdf.

[18] Møller H , Gildea C, Meechan D, Rubin G, Round T, Vedsted P. Use of the English urgent referral pathway for suspected cancer and mortality in patients with cancer: cohort study. BMJ 2015. 351:h5102. 26462713 10.1136/bmj.h5102 PMC4604216

[19] Food and Drug Administration . 24 Hour summary molecular and clinical genetics panel advisory committee meeting [Internet]; 2023. [cited 2024 Jan 22]. Available from: https://www.fda.gov/media/174413/download.

[20] Islami F , Goding Sauer A, Miller KD, Siegel RL, Fedewa SA, Jacobs EJ, . Proportion and number of cancer cases and deaths attributable to potentially modifiable risk factors in the United States: potentially preventable cancers in US. CA Cancer J Clin 2018;68:31–54. 29160902 10.3322/caac.21440

[21] Sewell B , Jones M, Gray H, Wilkes H, Lloyd-Bennett C, Beddow K, . Rapid cancer diagnosis for patients with vague symptoms: a cost-effectiveness study. Br J Gen Pract 2020;70:e186–92. 31932296 10.3399/bjgp20X708077 PMC6960004

[22] Frebourg T , Bajalica Lagercrantz S, Oliveira C, Magenheim R, Evans DG; European Reference Network GENTURIS. Guidelines for the Li–Fraumeni and heritable TP53-related cancer syndromes. Eur J Hum Genet 2020;28:1379–86. 32457520 10.1038/s41431-020-0638-4 PMC7609280

[23] Ballinger ML , Best A, Mai PL, Khincha PP, Loud JT, Peters JA, . Baseline surveillance in Li–Fraumeni syndrome using whole-body magnetic resonance imaging: a meta-analysis. JAMA Oncol 2017;3:1634–9. 28772291 10.1001/jamaoncol.2017.1968 PMC5824277

[24] Dolly SO , Jones G, Allchorne P, Wheeler D, Ali S, Mukadam Y, . The effectiveness of the Guy’s Rapid Diagnostic Clinic (RDC) in detecting cancer and serious conditions in vague symptom patients. Br J Cancer 2021;124:1079–87. 33402736 10.1038/s41416-020-01207-7 PMC7783491

[25] Arleo EK , Hendrick RE, Helvie MA, Sickles EA. Comparison of recommendations for screening mammography using CISNET models. Cancer 2017;123:3673–80. 28832983 10.1002/cncr.30842

[26] Croswell JM , Kramer BS, Kreimer AR, Prorok PC, Xu J-L, Baker SG, . Cumulative incidence of false-positive results in repeated, multimodal cancer screening. Ann Fam Med 2009;7:212–22. 19433838 10.1370/afm.942 PMC2682972

[27] Bredno J , Venn O, Chen X, Freese P, Ofman JJ. Circulating tumor DNA allele fraction: a candidate biological signal for multicancer early detection tests to assess the clinical significance of cancers. Am J Pathol 2022;192:1368–78. 35948080 10.1016/j.ajpath.2022.07.007

[28] Chen X , Dong Z, Hubbell E, Kurtzman KN, Oxnard GR, Venn O, . Prognostic significance of blood-based multi-cancer detection in Plasma cell-free DNA. Clin Cancer Res 2021;27:4221–9. 34088722 10.1158/1078-0432.CCR-21-0417 PMC9401481

[29] Patel A , Clarke CA, Deubler EL, Fung ET, Jiang R, Lichtman C, . Preclinical circulating tumor DNA (ctDNA) shedding duration and prognostic implications of modeling 3669 participants from American Cancer Society Cancer Prevention Study-3 (CPS-3) and Circulating Cell-free Genome Atlas substudy 3 (CCGA3). J Clin Oncol 2023;41:3060. 10.1002/cncr.70380 PMC13063101 41953965

[30] Swanton C , Margolis M, Hubbell E, Kurtzman KN, Shih S, Venn O, . Abstract 3895: prognostic significance of blood-based multi-cancer detection in cell-free DNA: 4-year outcomes analysis. Cancer Res 2024;84:3895.

[31] Mahal BA , Margolis M, Hubbell E, Chen C, Venstrom JM, Abran J, . A targeted methylation–based multicancer early detection blood test preferentially detects high-grade prostate cancer while minimizing overdiagnosis of indolent disease. JCO Precis Oncol 2024;8:e2400269. 39208374 10.1200/PO.24.00269 PMC11371104

[32] Hudnut AG , Hubbell E, Venn O, Church TR. Modeled residual current cancer risk after clinical investigation of a positive multicancer early detection test result. Cancer 2023;129:2056–63. 36943898 10.1002/cncr.34747

[33] Neal RD , Johnson P, Clarke CA, Hamilton SA, Zhang N, Kumar H, . Cell-free DNA–based multi-cancer early detection test in an asymptomatic screening population (NHS-Galleri): design of a pragmatic, prospective randomised controlled trial. Cancers 2022;14:4818. 36230741 10.3390/cancers14194818 PMC9564213

[34] Dai JY , Georg Luebeck E, Chang ET, Clarke CA, Hubbell EA, Zhang N, . Strong association between reduction of late-stage cancers and reduction of cancer-specific mortality in meta-regression of randomized screening trials across multiple cancer types. J Med Screen 2024;31:211–22. 38797981 10.1177/09691413241256744 PMC11528850

[35] Lange JM , Gogebakan KC, Gulati R, Etzioni R. Projecting the impact of multi-cancer early detection on late-stage incidence using multi-state disease modeling. Cancer Epidemiol Biomarkers Prev 2024;33:830–7. 38506751 10.1158/1055-9965.EPI-23-1470 PMC11213491

[36] Etzioni R , Gulati R, Patriotis C, Rutter C, Zheng Y, Srivastava S, . Revisiting the standard blueprint for biomarker development to address emerging cancer early detection technologies, J Natl Cancer Inst 2024;116:189–93. 37941446 10.1093/jnci/djad227 PMC10852609

[37] Webb AB , Berg CD, Castle PE, Crosby D, Etzioni R, Kessler LG, . Considerations for using potential surrogate endpoints in cancer screening trials. Lancet Oncol 2024;25:e183–92. 38697164 10.1016/S1470-2045(24)00015-9 PMC7616115

[38] Nicholson BD , Oke J, Virdee PS, Harris DA, O’Doherty C, Park JE, . Multi-cancer early detection test in symptomatic patients referred for cancer investigation in England and Wales (SYMPLIFY): a large-scale, observational cohort study. Lancet Oncol 2023;24:733–43. 37352875 10.1016/S1470-2045(23)00277-2

[39] Verduzco-Aguirre HC , Lopes G, Soto-Perez-De-Celis E. Implementation of diagnostic resources for cancer in developing countries: a focus on PET/CT. Ecancermedicalscience 2019 13:ed87. 30915165 10.3332/ecancer.2019.ed87 PMC6390832

[40] Hanna TN , Friedberg E, Dequesada IM, Chaves L, Pyatt R, Duszak R, . Disparities in the use of emergency department advanced imaging in medicare beneficiaries, AJR Am J Roentgenol 2021;216:519–25. 33356434 10.2214/AJR.20.23161

[41] Quinn B , Dauer Z, Pandit-Taskar N, Schoder H, Dauer LT. Radiation dosimetry of 18F-FDG PET/CT: incorporating exam-specific parameters in dose estimates. BMC Med Imaging 2016;16:41. 27317478 10.1186/s12880-016-0143-y PMC4912712

[42] Westgate C , Gordon O, Margolis M, Oh Y, Broyles D, Friedman J, . 1184P Early real-world experience with positive multi-cancer early detection (MCED) test cases and negative initial diagnostic work-up. Ann Oncol 2024;35:S766–7.

[43] Xu DM , Gietema H, de Koning H, Vernhout R, Nackaerts K, Prokop M, . Nodule management protocol of the NELSON randomised lung cancer screening trial. Lung Cancer 2006;54:177–84. 16989922 10.1016/j.lungcan.2006.08.006

[44] de Koning HJ , van der Aalst CM, de Jong PA, Scholten ET, Nackaerts K, Heuvelmans MA, . Reduced lung-cancer mortality with volume CT screening in a randomized trial. N Engl J Med 2020;382:503–13. 31995683 10.1056/NEJMoa1911793

