# Supplementary material for: Modeled Benefit of Individual Cancer Signal Origin Prediction for Multi-Cancer Early Detection
Source: Cancer Res Commun. 2025 May 19;5(5):814–24. doi: 10.1158/2767-9764.CRC-24-0351 (PMC12087281; doi:10.1158/2767-9764.CRC-24-0351)
Supplement: Supplementary Materials — Supplementary Methods, Data, and References [file crc-24-0351_supplementary_materials_suppsm.docx]

## Supplementary Materials

### Supplementary Methods

**Brief description of the interception model**

In the interception model [(1)](https://www.zotero.org/google-docs/?a2rCUO), it is assumed that cancer develops in an individual from a non-cancer state, proceeds progressively through preclinical stages I-IV, and may be detected either clinically or by an MCED test in these stages. Detection by MCED test is possible when a cancer starts shedding detectable quantities of DNA and is reliable after that point. That point at which shedding becomes detectable may occur at any stage, even at stage IV, or never occur at all. We can therefore treat the cancer cases as proceeding on one of five tracks in a state-transition model depending on the stage within which it becomes detectable (**Supplementary Figure 1**).

The model is calibrated by noting the rate of clinical diagnosis in SEER at each stage, as well as the shedding rate of DNA by cancers at that stage. This determines the proportion of individuals with cancer shedding DNA at each stage, and hence the relative proportions of cases proceeding along each track. Interception by an MCED test occurs when a cancer case is detectable, and a blood draw occurs within the dwell time for that stage. The effect is to divert detectable cases to MCED test detection at a stage before or equal to clinical diagnosis, at a rate dependent on the frequency of blood draw vs the distribution of dwell times.

We exploit the stability of incidence screening rounds to compute the final stable state of screening in long term behavior by noting that the total incidence of cancer must remain the same after screening (in the absence of overdiagnosis and in the absence of removal of precursors of cancer), so the final incidence in any year of screening is divided between MCED test detection and clinical detection in a constant manner.

Computations of the cancers intercepted by MCED are done by numerically integrating over the dwell time distributions to determine what proportion of cases are intercepted at each stage given a screening interval. Each cancer type is computed in parallel using the detection rates for that cancer type by stage, as well as the dwell times assigned to that cancer type.

False positives are generated among the non-cancers at a rate determined by the specificity. Note that false positives do not have a cancer type and their rate of occurrence does not depend on the number of cancer types screened.

All cancer signal detected cases have a cancer signal origin to guide workup assigned to them by a specific cancer signal origin classifier. True positives are likely to receive a cancer signal matching the expected cancer signal origin; false positives have no cancer type and therefore no expected cancer signal origin and may receive any cancer signal origin (see discussion of cancer signal origin classifier below).

**Behavior of the cancer signal origin classifier and frequency estimates**

The cancer signal origin (CSO) classifier is designed to output a cancer signal origin prediction for a cancer signal detected result. It is therefore trained on a set of cancer cases that received cancer signal detected results, or would receive a cancer signal detected result at a slightly lower specificity. Each training case has a clinical cancer signal origin assigned based on the cancer type. The probability distribution of clinical cancer signal origin predictions in the training data is therefore similar to the probability distribution of clinical cancer signal origin predictions in those receiving a cancer signal detected result. This distribution is very different from the distribution of cancers and associated clinical cancer signal origin predictions in the total training data. Because the cancer signal origin classifier has been trained to high accuracy, significant systematic deviations from this distribution could be accounted for and used to improve classification, and so are unlikely to occur.

We therefore approximate the default probability distribution for cancer signal origin prediction by this probability distribution. This default distribution is used as a prior distribution to fill in the relative probabilities of unobserved events. The validation set contains many observations of cancer cases with a cancer signal detected, and so the prior is given a small weight when used to augment true positives. Because of the low absolute number of false positives observed in the validation set due to the high specificity, the prior is given a large weight when used to augment false positives. This may be thought of as a Bayesian posterior probability given the observed entries in the validation set. Cancer signal origin predictions incompatible with a given sex are suppressed, with the probability mass in forbidden outcomes redistributed proportionately to the allowed cancer signal origin predictions.

**Shared cancer signal and uncommon (including rare) cancers**

MCED tests, including the one currently available [(2)](https://www.zotero.org/google-docs/?h8DtOv), may detect common features shared across many cancers, and therefore affect the continuum of care of a broad spectrum of cancers. According to this model, the potential conditional benefit of each predicted cancer signal origin is enough to warrant specific, single-cancer directed diagnostic workup for any cancer signal origin prediction, including those representing uncommon (including individually rare) cancers. Further, even if site-directed diagnostic tests are effectively perfect and allow for ruling out a specific cancer, there is often sufficient evidence that justifies a general diagnostic test in the remaining individuals. In the real world, with imperfect diagnostic performance, continuing to evaluate cancer status in individuals receiving a cancer signal detected result is important. Analyzed in the context of all cancers, our model suggests that cancer signal origin predictions for any cancer, including less common ones, should be considered for workup because individuals do not choose which cancers they may develop. This is reflected in such statistics that individually uncommon (including rare) cancers account for more than 20% of diagnoses and deaths [(3,4)](https://www.zotero.org/google-docs/?PrerNN), collectively of similar importance as lung cancer. Neglecting this large segment of cancer mortality reduces the clinical potential of an MCED test to affect cancer mortality.

**Exclusions from modelable cancer signal origins**

Some cancer signal origins are difficult or impossible to model for some quantities due to limits of the data and are excluded from reporting, although present in data tables and figures for completeness. Thyroid cancers had no detections reported in CCGA3, and therefore we cannot model any detection events occurring for PPV or lives saved. Myeloid Neoplasm had no successful predictions of cancer signal origin and so PPV for CSO-directed workups appears very low (on very limited data - two detections), and is unstaged, so cannot be modeled for lives saved. Melanoma does not have any detections reported at stages I-III, so no changes in stage distribution are modelable, which results in no lives saved being modelable. Finally, Plasma Cell Neoplasm has no staged survival data in SEER, which means that changes in stage distribution cannot be estimated, nor can lives saved be estimated.

Cancers with no staging included in cancer signal origins (Lymphoid Neoplasm) also cannot have lives saved estimated, although they are included in cancers detected. In general, this means that lives saved is an underestimate of the potential gain from detection within each cancer signal origin.

**Sensitivity analysis for stochastic variability**

Sensitivity analysis for stochastic variability in the input data was done using repeated draws from the posterior distribution for sensitivity per cancer and the posterior distribution for false positive rates given the observed counts in CCGA3. This is a draw from a beta distribution with a Jeffreys prior (noninformative). For sensitivity, the usual weighted pool-adjacent violators algorithm, a form of smoothing to create monotonicity, is used to generate non-decreasing estimates by stage from the posterior draws.

Uncertainty in the cancer signal origin classifier results is handled similarly, using a Dirichlet-multinomial distribution from the observed counts with an uninformative reference prior.

Alternate results of stochastic simulation matching those presented via Figures 2-6 of the main text are provided below as **Supplementary Figures 2-6**, which plot median and confidence intervals based on uncertainty in sensitivity, specificity, and cancer signal origin assignment. As noted in the main text, the primary driver of this uncertainty is the stochastic variation in the false positive rate. A secondary source of variation can be seen for various cancers where there is little evidence for detection, accurate cancer signal origin prediction, or lives saved in the original data (thyroid, melanoma, myeloid), due to lack of sensitivity, or inadequate sampling of cancer signal origin predictions, but including the possibility of nonzero sensitivity at earlier stages yields some potential benefits.

A further pattern can be seen by observing that both PPV and lives saved per diagnostic tests are related in different ways to the number of diagnostic tests, which is driven primarily by false positive rates vs incidence. We can show these values across all iterations (ages 55, 65, 75, and exposure levels and sexes) and observe that generally, when PPV is above 7%, lives saved is reasonable; even when PPV is below 7%, lives saved per diagnostic test is still better than mammography (240) (**Supplementary Figures 7-8**).

Cases where items fall outside the specified ranges are generally as expected, eg, in the lower age range where incidence is low compared to false positive rates, although they are in the minority.

We omit here the cases where modeling is not generally informative for both quantities: Plasma Cell Neoplasm and Myeloid Neoplasm cancer signal origins lack stage information and so lives saved cannot be modeled; Thyroid and Melanocytic lineage cancer signal origins lack sufficient sensitivity information at early stage to reliably estimate stage shift and hence lives saved; and male breast cancer (Breast cancer signal origin in males) has low incidence and few examples in the training data.

**Sensitivity analysis of dwell times**

Following Hubbell et al (2021) [(1)](https://www.zotero.org/google-docs/?lmqb2q), we compute two different dwell time scenarios: “Fast” (dwell times ~ 2 years) and “Fast Aggressive” (dwell times ~ 1 year). The practical effect is to lower the episode sensitivity as the dwell time decreases by increasing the fraction of interval cancers, which reduces the PPV of positive tests. A second (minor) effect is to alter the stage distribution of those found through screening, which alters lives saved. We concentrate here on faster dwell times other than the default scenario because we are most interested in guarding against potential decreases in performance.

We illustrate with two figures comparing a particular example as in the main text, showing how PPV and lives saved each shift by dwell time. These show very minimal shifts in performance driven by dwell time alone within this range. (**Supplementary Figures 9-10**)

Summarizing a large number of individual data points across all population situations: for cases where we can model both lives saved and PPV, there are a few cases where the PPV for CSO-directed testing is below the 7% threshold, concentrated in young never smokers (for Lung and Neuroendocrine cancer signal origins), but these do not significantly vary between dwell scenarios and have sufficient lives saved if pursued. More cases occur in post-CSO-directed additional testing with lowered PPV; but again, they are largely similar between scenarios and generally have high lives saved per diagnostic test. These cases are unsurprisingly concentrated in younger ages and never smokers, where lower incidence of all cancer occurs. (**Supplementary Figures 11-12**)

Full data files are available in the Supplementary Materials.

**Differences between OS and CSS for lives saved computations**

Lives saved computations in the main manuscript use cancer-specific survival (CSS), as we are typically concerned with individuals with sufficient life remaining that competing risks do not overly reduce the effect of screening. While this is a potential issue at older ages, the diagnostic tests needed to save a life has a considerable variability among cancer types and compares favorably to the average for mammography (240), even at older ages. This holds generally for CSO-directed tests, and similarly for general diagnostic tests done after CSO-directed tests to account for residual risk of cancer. Note that at younger ages, due to the lower incidence of cancer compared to false positives under this model, there is a relative increase in diagnostic tests required, which is larger than the increase due to competing mortality at older ages. (**Supplementary Figures 13-14**)

At the lower boundary of typical screening ages (50 years), diagnostic tests per life saved exceeds the default limit for female cervix post-CSO-directed testing (because cancer signal origin prediction is quite accurate for cervix with little expected cross-talk); similarly, male anal cancer (age 50 years) exceeds the default. These are exceptions, as most cases have good tradeoffs.

Again, here we limit to cases that are modelable for lives saved with reliability.

**Sensitivity analysis: Increased hazard for cell-free DNA (cfDNA) positive cancer cases**

The subset of cancers not shedding cfDNA may contain relatively indolent subsets of cancer cases, reducing the hazard for cfDNA-negative cases and increasing hazard for cfDNA-positive cases to maintain the average survival hazard. This concentrates cancer-specific death into cases that might be detected by an MCED screening technology using cfDNA. Under this model, there are a mixture of effects - beneficial by sweeping more deaths into the subset of cases that can be affected by early detection, but reduces the potential benefit to early detection. We increase here the relative hazard ratio for cfDNA-positive vs cfDNA-negative to 2 (maintaining the average survival, based on the fraction cfDNA-positive and negative).

This only affects lives saved computations. Plotted below are typical lives saved values with original cancer-specific survival vs increased hazard for both CSO-directed and post-CSO-directed analyses. As in other analyses, the effect is small relative to the chosen threshold (mammography). (**Supplementary Figures 15-16**)

**Strategies avoiding use of cancer signal origin information**

As noted in the Introduction, cancer signal origin information is compatible with existing medical care, where focused investigation using least invasive procedures is the norm, as well as specifically being called out by the FDA as a needed component of an MCED report. However, physicians may choose to ignore cancer signal origin information and use maximal investigative techniques, such as whole body PET-CT.

This strategy can be simply recovered by averaging out over all cancer signal origin information. Note that under the default strategy, some non-CSO-directed tests are required because of the residual risk of cancer after CSO-directed testing. These non-CSO-directed tests are largely required to eliminate false positives (however, see Discussion about alternate means of false positive elimination). The number of such general tests is always smaller than the number required when avoiding use of CSO-directed information. While the total number of tests is larger under a CSO-directed strategy, it is not an undue burden given the low number of false positives. (**Supplementary Figure 17**)

We can further note the ratio between additional CSO-directed and additional non-CSO-directed tests as an approximate estimate of tradeoffs between such strategies (**Supplementary Figure 18**); however, precise costs are not available and are out of scope for this modeling paper.

**Strategies only using CSO-directed workups**

It is possible that whole-body imaging or other non-CSO-directed diagnostic workups are unavailable or not thought to be worthwhile given the residual risk. Such strategies forego additional testing, which misses cancer cases where outcomes may be improved. Modeling such one-and-done strategies is simply done by omitting further workups. We see that the fraction of potential lives saved in such a strategy remains high because the high accuracy of cancer signal origin prediction leads to most true cancer cases being found. (**Supplementary Figure 19**)

However, the absolute residual risk of cancer remains high for individuals of older ages who are at high absolute risk of cancer before testing. We show this residual risk and the age at which this rises above the threshold of 7%. (**Supplementary Figure 20**)

### Supplementary Data

Supplementary Table 1 is a superset of the results plotted in the figures. It shows the performance in “incidence rounds” of screening, which models the long-term performance of a screening program. Such rounds are when most cancers will be detected and have the greatest decrease in late-stage incidence.

Supplementary Table 2 shows the “prevalence round” of screening, ie, the first round of screening in which many individuals will have existing undiagnosed cancers because there will have been no prior round of screening in which those cancers could have been found early. These tables can be accessed from the Excel spreadsheet at <https://github.com/grailbio-publications/Klein_CSO_Benefit>.

### Supplementary References

[1. Hubbell E, Clarke CA, Aravanis AM, Berg CD. Modeled Reductions in Late-stage Cancer with a Multi-Cancer Early Detection Test. Cancer Epidemiol Biomarkers Prev. 2021;30:460–8.](https://www.zotero.org/google-docs/?RG5aJF)

[2. Klein E, Richards D, Cohn A, Tummala M, Lapham R, Cosgrove D, et al. Clinical validation of a targeted methylation-based multi-cancer early detection test using an independent validation set. Ann Oncol. Elsevier; 2021;32:1167–77.](https://www.zotero.org/google-docs/?RG5aJF)

[3. Gatta G, Capocaccia R, Botta L, Mallone S, De Angelis R, Ardanaz E, et al. Burden and centralised treatment in Europe of rare tumours: results of RARECAREnet—a population-based study. Lancet Oncol. 2017;18:1022–39.](https://www.zotero.org/google-docs/?RG5aJF)

[4. Botta L, Gatta G, Trama A, Bernasconi A, Sharon E, Capocaccia R, et al. Incidence and survival of rare cancers in the US and Europe. Cancer Med. 2020;9:5632–42.](https://www.zotero.org/google-docs/?RG5aJF)
