# Supplementary material for: Modeled Benefit of Individual Cancer Signal Origin Prediction for Multi-Cancer Early Detection
Source: Cancer Res Commun. 2025 May 19;5(5):814–24. doi: 10.1158/2767-9764.CRC-24-0351 (PMC12087281; doi:10.1158/2767-9764.CRC-24-0351)

**Supplementary Figure 1.** State transition diagram for the interception model, expanded to show the five possible trajectories of cancer detectability by stage, and the potential detection by MCED or by usual care.


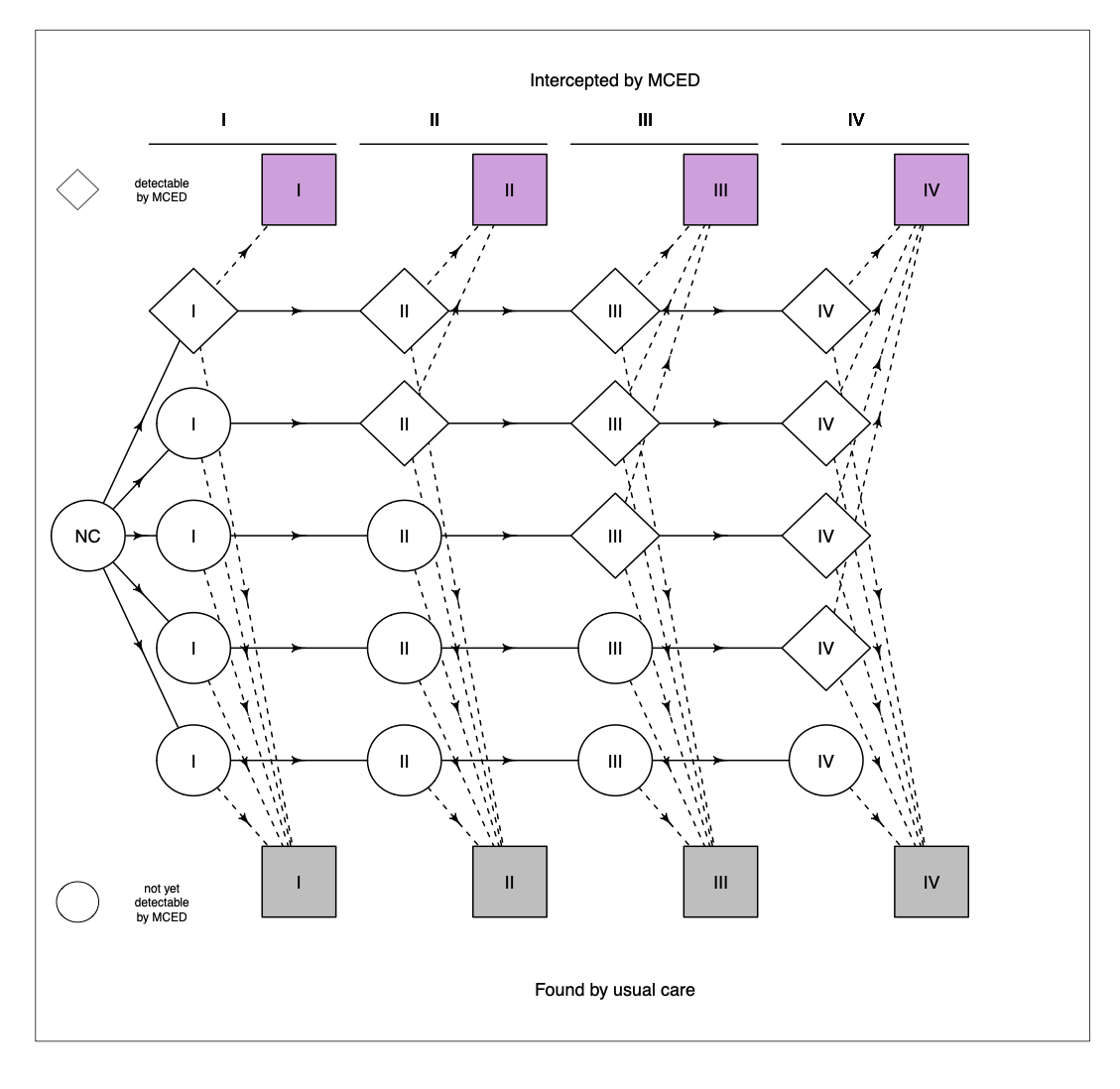

Supplement: Supplementary Figure 1 — State transition diagram for the interception model, expanded to show the five possible trajectories of cancer detectability by stage, and the potential detection by MCED or by usual care [file crc-24-0351_supplementary_figure_1_suppsf1.docx]
