# Supplementary material for: Modeled Benefit of Individual Cancer Signal Origin Prediction for Multi-Cancer Early Detection
Source: Cancer Res Commun. 2025 May 19;5(5):814–24. doi: 10.1158/2767-9764.CRC-24-0351 (PMC12087281; doi:10.1158/2767-9764.CRC-24-0351)

**Supplementary Figure 5**. All PPVs in the modeled diagnostic chain for 65- to 69-year-old females with any smoking status, accounting for uncertainty in sensitivity, specificity, and cancer signal origin assignment. The dashed line represents a threshold PPV of 7% typically justifying workup.


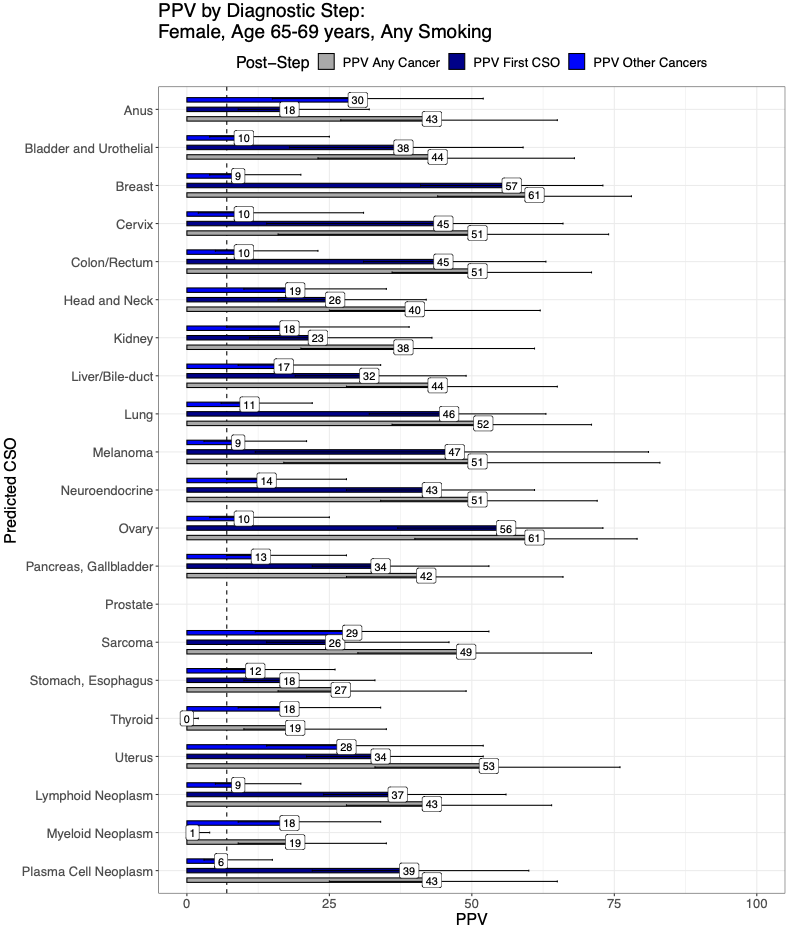

Supplement: Supplementary Figure 5 — All PPVs in the modeled diagnostic chain for 65- to 69-year-old females with any smoking status, accounting for uncertainty in sensitivity, specificity, and cancer signal origin assignment [file crc-24-0351_supplementary_figure_5_suppsf5.docx]
