# Supplementary material for: Modeled Benefit of Individual Cancer Signal Origin Prediction for Multi-Cancer Early Detection
Source: Cancer Res Commun. 2025 May 19;5(5):814–24. doi: 10.1158/2767-9764.CRC-24-0351 (PMC12087281; doi:10.1158/2767-9764.CRC-24-0351)

**Supplementary Figure 6**. Number of 65- to 69-year-old females with any smoking history who need to undergo a diagnostic test to save one life for each cancer signal origin, accounting for uncertainty in sensitivity, specificity, and cancer signal origin assignment. The dashed line represents a modeled benchmark of 240 diagnostic mammograms needed to save one life.


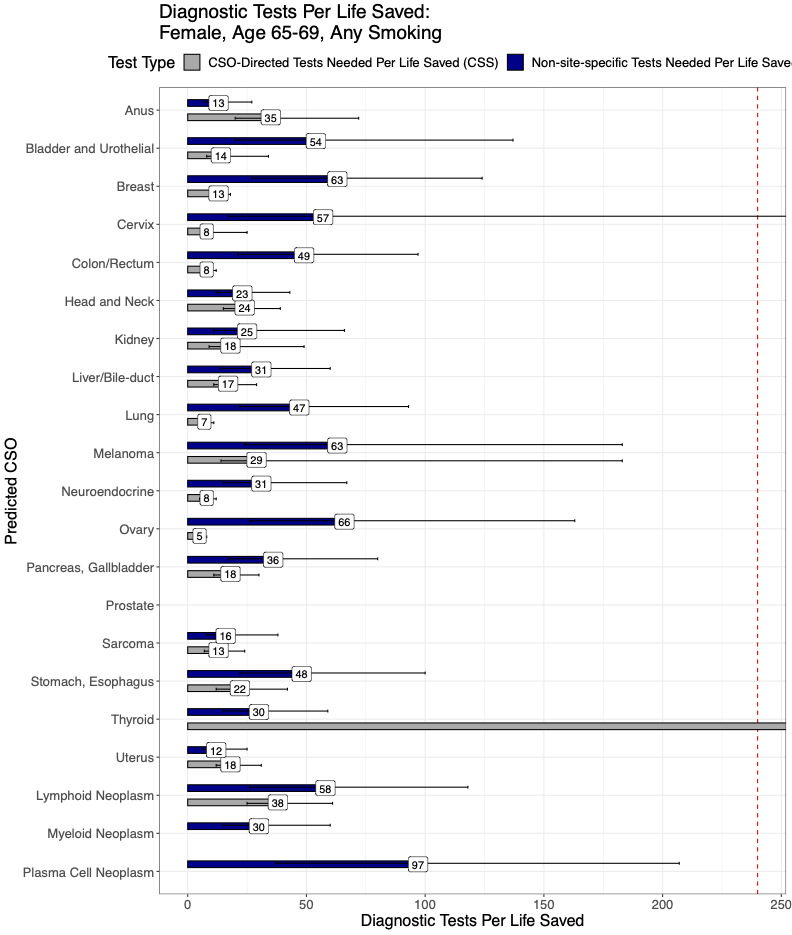

Supplement: Supplementary Figure 6 — Number of 65- to 69-year-old females with any smoking history who need to undergo a diagnostic test to save one life for each cancer signal origin, accounting for uncertainty in sensitivity, specificity, and cancer signal origin assignment [file crc-24-0351_supplementary_figure_6_suppsf6.docx]
