# Supplementary material for: Modeled Benefit of Individual Cancer Signal Origin Prediction for Multi-Cancer Early Detection
Source: Cancer Res Commun. 2025 May 19;5(5):814–24. doi: 10.1158/2767-9764.CRC-24-0351 (PMC12087281; doi:10.1158/2767-9764.CRC-24-0351)

**Supplementary Figure 7**: Relationship between PPV and diagnostic tests to save a life for CSO-directed workups, shown stratified by cancer signal origin, colored by sex. All draws from the stochastic model are plotted here, as well as age bands covering 50-79 years. Dashed lines reflect the 7% PPV comparison and the 240 diagnostic tests per live saved comparison. Note that even when PPV is below the comparison line, lives saved are still favorable.


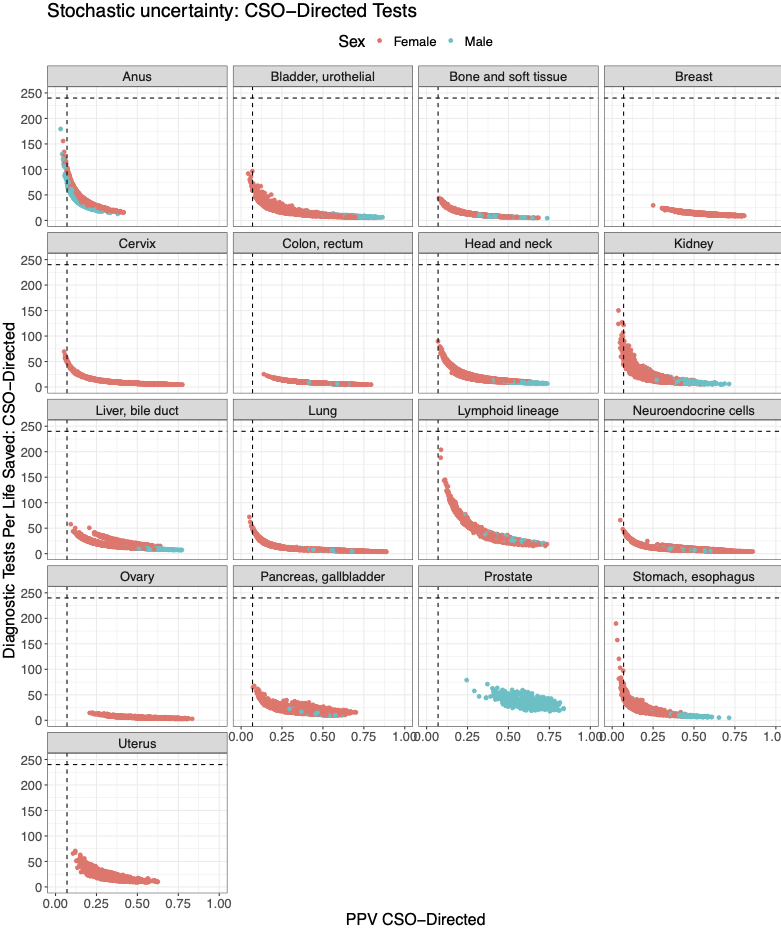

Supplement: Supplementary Figure 7 — Relationship between PPV and diagnostic tests to save a life for CSO-directed workups, shown stratified by cancer signal origin, colored by sex [file crc-24-0351_supplementary_figure_7_suppsf7.docx]
