# Supplementary material for: Modeled Benefit of Individual Cancer Signal Origin Prediction for Multi-Cancer Early Detection
Source: Cancer Res Commun. 2025 May 19;5(5):814–24. doi: 10.1158/2767-9764.CRC-24-0351 (PMC12087281; doi:10.1158/2767-9764.CRC-24-0351)

**Supplementary Figure 8:** Relationship between PPV and diagnostic tests to save a life for post-CSO-directed workups, shown stratified by cancer signal origin, colored by sex. All draws from the stochastic model are plotted here, as well as age bands covering 50-79 years. Dashed lines reflect the 7% PPV comparison and the 240 diagnostic tests per live saved comparison. Note that even when PPV is below the comparison line, lives saved are often still favorable. Some cases, as noted in text, fail both comparison metrics. For example, individuals with a Cervix cancer signal origin who do not have cervical cancer are sufficiently rare in younger age ranges (due to a combination of low incidence and high accuracy cancer signal origin prediction) so that it may be worthwhile terminating workup after a CSO-directed workup without any post-CSO general workups.


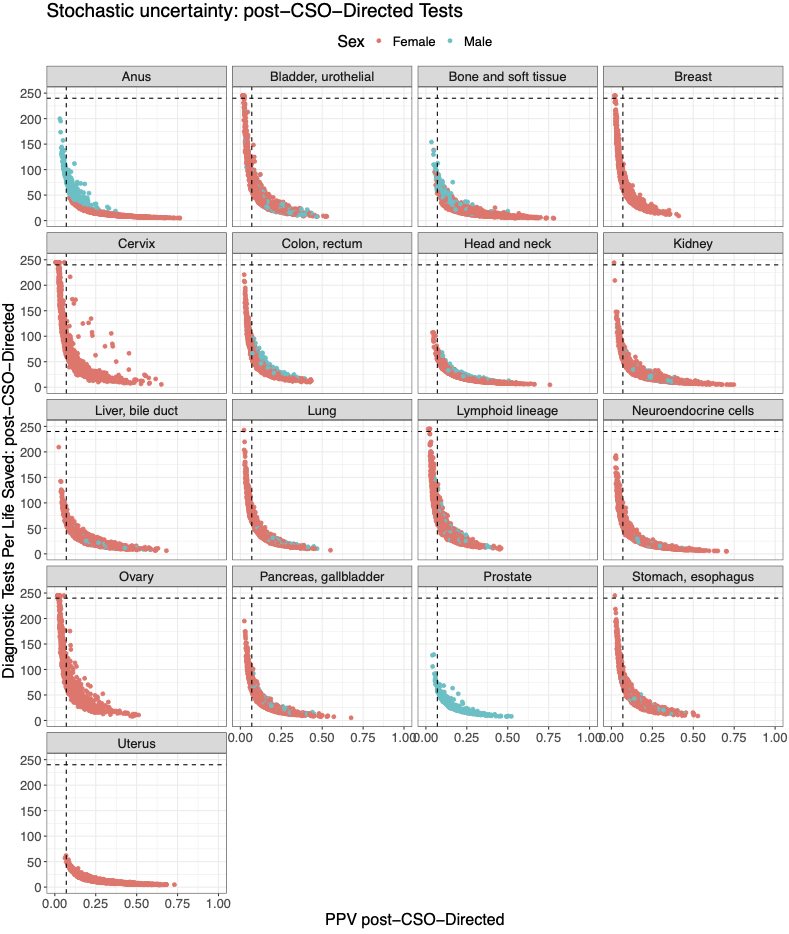

Supplement: Supplementary Figure 8 — Relationship between PPV and diagnostic tests to save a life for post-CSO-directed workups, shown stratified by cancer signal origin, colored by sex [file crc-24-0351_supplementary_figure_8_suppsf8.docx]
