# Supplementary material for: Modeled Benefit of Individual Cancer Signal Origin Prediction for Multi-Cancer Early Detection
Source: Cancer Res Commun. 2025 May 19;5(5):814–24. doi: 10.1158/2767-9764.CRC-24-0351 (PMC12087281; doi:10.1158/2767-9764.CRC-24-0351)

**Supplementary Figure 9:** Overall PPV for any cancer, stratified by cancer signal origin with comparison across dwell time scenarios. Because annual screening is still comparable to the total time cancers spend detectable, detection rates of cancer do not drop significantly.


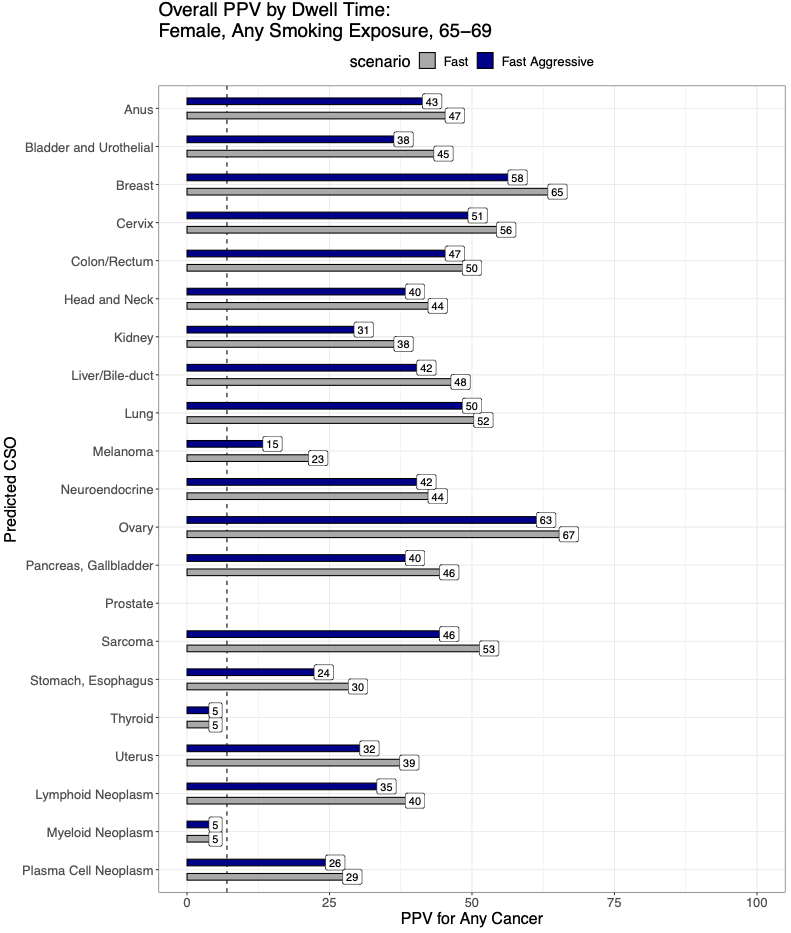

Supplement: Supplementary Figure 9 — Overall PPV for any cancer, stratified by cancer signal origin with comparison across dwell time scenarios [file crc-24-0351_supplementary_figure_9_suppsf9.docx]
