# Supplementary material for: Modeled Benefit of Individual Cancer Signal Origin Prediction for Multi-Cancer Early Detection
Source: Cancer Res Commun. 2025 May 19;5(5):814–24. doi: 10.1158/2767-9764.CRC-24-0351 (PMC12087281; doi:10.1158/2767-9764.CRC-24-0351)

**Supplementary Figure 11:** PPV plotted against diagnostic tests per lives saved across age ranges and sexes, stratified by dwell time scenario. Again, we see that small overall shifts due to dwell times changing do not take most cases outside of clinically actionable ranges.


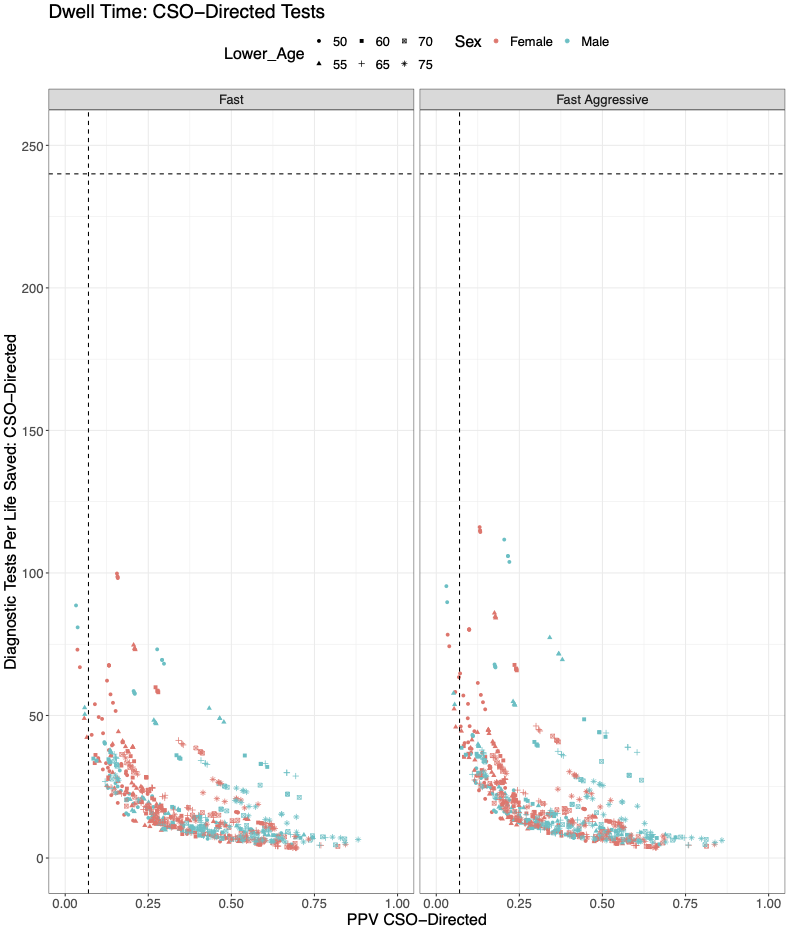

Supplement: Supplementary Figure 11 — PPV plotted against diagnostic tests per lives saved across age ranges and sexes, stratified by dwell time scenario [file crc-24-0351_supplementary_figure_11_suppsf11.docx]
