# Supplementary material for: Modeled Benefit of Individual Cancer Signal Origin Prediction for Multi-Cancer Early Detection
Source: Cancer Res Commun. 2025 May 19;5(5):814–24. doi: 10.1158/2767-9764.CRC-24-0351 (PMC12087281; doi:10.1158/2767-9764.CRC-24-0351)

**Supplementary Figure 10:** Diagnostic tests per life saved for all cancer signal origins, comparison across dwell time scenarios. Because cancers that are found are still likely to benefit in terms of lives saved, changes in this metric are primarily driven by changes in PPV. The plotted scenario corresponds to Figure 6 in the main manuscript, but only shows CSO-directed tests to illustrate.


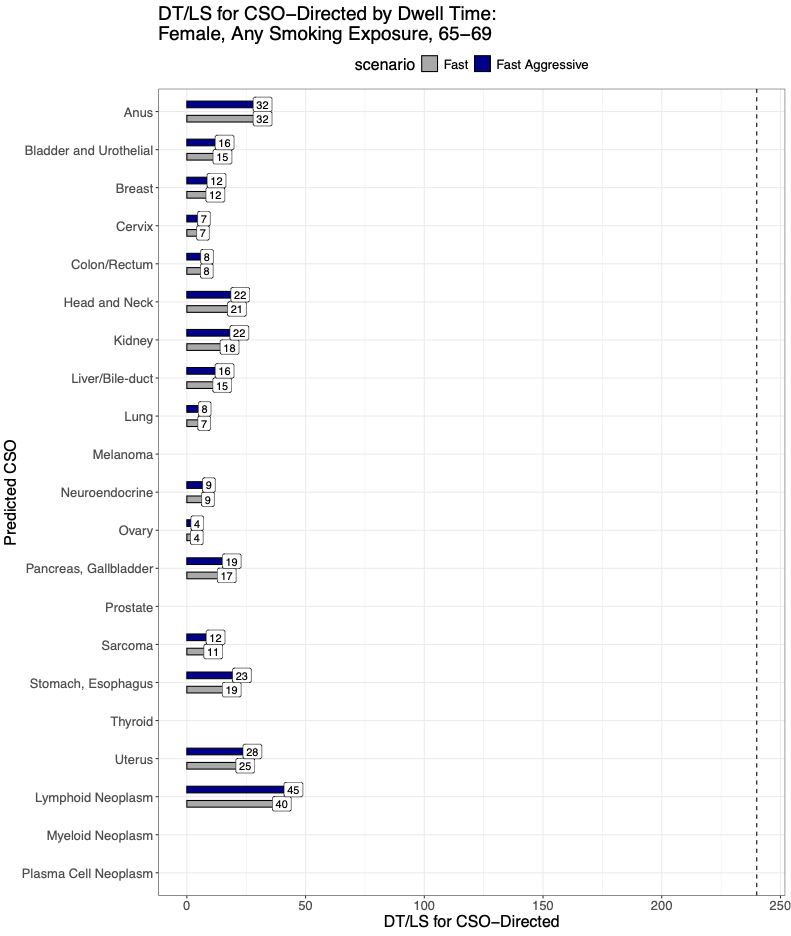

Supplement: Supplementary Figure 10 — Diagnostic tests per life saved for all cancer signal origins, comparison across dwell time scenarios [file crc-24-0351_supplementary_figure_10_suppsf10.docx]
