# Supplementary material for: Modeled Benefit of Individual Cancer Signal Origin Prediction for Multi-Cancer Early Detection
Source: Cancer Res Commun. 2025 May 19;5(5):814–24. doi: 10.1158/2767-9764.CRC-24-0351 (PMC12087281; doi:10.1158/2767-9764.CRC-24-0351)

**Supplementary Figure 12:** PPV plotted against diagnostic tests per lives saved, comparison between dwell time scenarios for post-CSO-directed workups. Younger ages again may drop below 7% PPV but still retain an actionable level of diagnostic tests per lives saved.


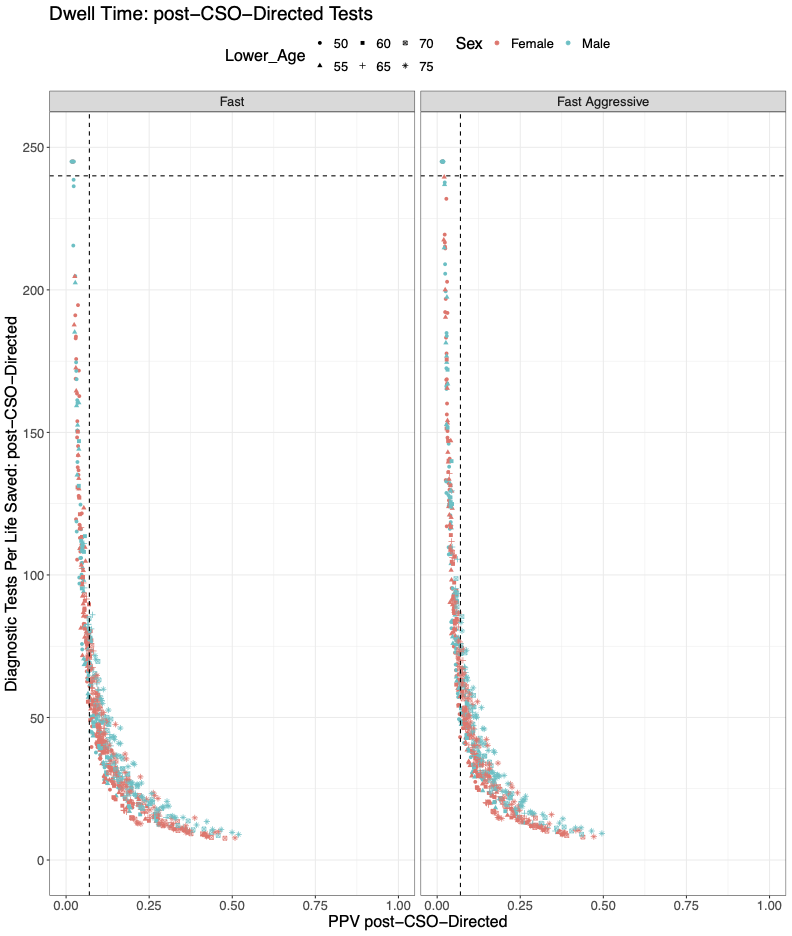

Supplement: Supplementary Figure 12 — PPV plotted against diagnostic tests per lives saved, comparison between dwell time scenarios for post-CSO-directed workups [file crc-24-0351_supplementary_figure_12_suppsf12.docx]
