# Supplementary material for: Modeled Benefit of Individual Cancer Signal Origin Prediction for Multi-Cancer Early Detection
Source: Cancer Res Commun. 2025 May 19;5(5):814–24. doi: 10.1158/2767-9764.CRC-24-0351 (PMC12087281; doi:10.1158/2767-9764.CRC-24-0351)

**Supplementary Figure 13:** Diagnostic tests per lives saved for CSO-directed workups, age bands covering 50-80 years, incidence as default for SEER (“any” smoking status as smoking status is unknown in SEER). CSS and OS differences in this estimate are shown as boxplots containing all cancer signal origins. For younger ages, the two are very similar because competing risks are low and, while there is a shift on average at older ages, they are all within a clinically actionable range. This is in part because the increased cancer incidence at older ages leads to fewer diagnostic tests required per life saved, counterbalancing some of the competing risk, which reduces lives saved.


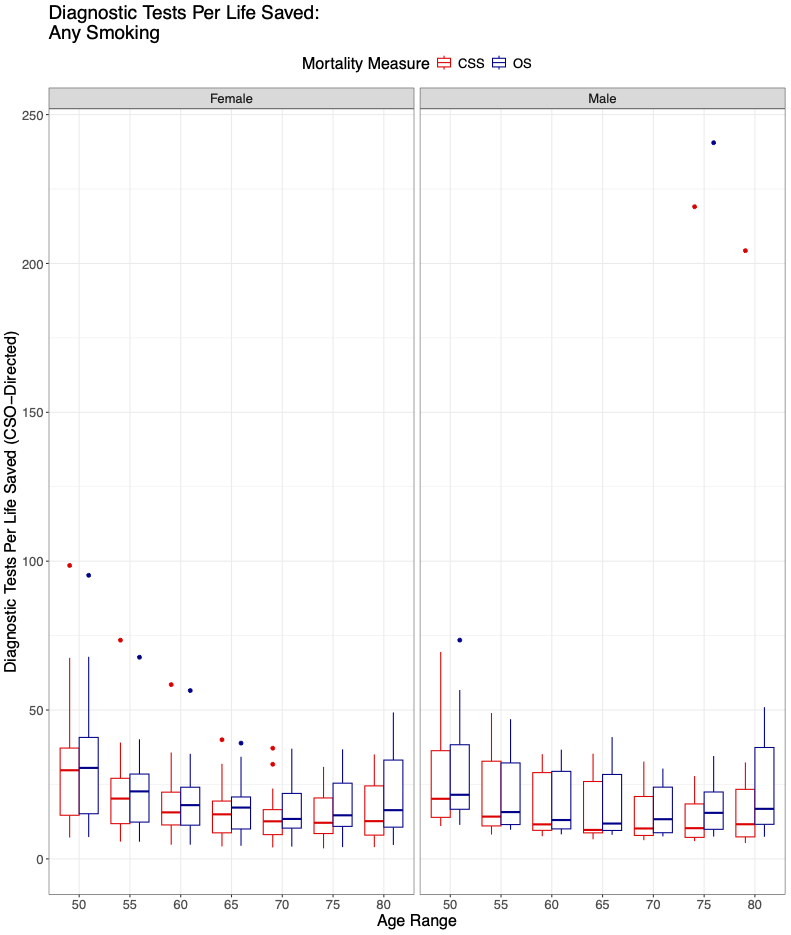

Supplement: Supplementary Figure 13 — Diagnostic tests per lives saved for CSO-directed workups, age bands covering 50-80 years, incidence as default for SEER (“any” smoking status as smoking status is unknown in SEER) [file crc-24-0351_supplementary_figure_13_suppsf13.docx]
