# Supplementary material for: Modeled Benefit of Individual Cancer Signal Origin Prediction for Multi-Cancer Early Detection
Source: Cancer Res Commun. 2025 May 19;5(5):814–24. doi: 10.1158/2767-9764.CRC-24-0351 (PMC12087281; doi:10.1158/2767-9764.CRC-24-0351)

**Supplementary Figure 14:** Diagnostic tests per lives saved for post-CSO-directed workups, age bands covering 50-80 years, incidence as default for SEER (“any” smoking status as smoking status is unknown in SEER). CSS and OS differences in this estimate are shown as boxplots containing all cancer signal origins. For younger ages, the two are very similar because competing risks are low and, while there is an increase at older ages, they are generally within clinically actionable range.


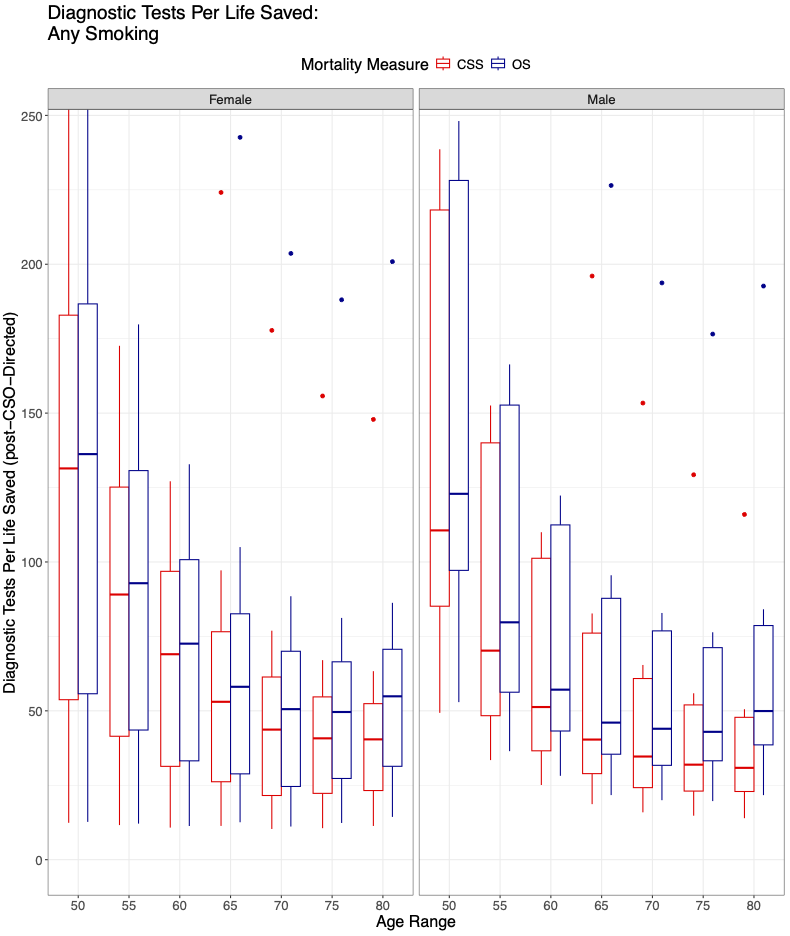

Supplement: Supplementary Figure 14 — Diagnostic tests per lives saved for post-CSO-directed workups, age bands covering 50-80 years, incidence as default for SEER [file crc-24-0351_supplementary_figure_14_suppsf14.docx]
