# Supplementary material for: Modeled Benefit of Individual Cancer Signal Origin Prediction for Multi-Cancer Early Detection
Source: Cancer Res Commun. 2025 May 19;5(5):814–24. doi: 10.1158/2767-9764.CRC-24-0351 (PMC12087281; doi:10.1158/2767-9764.CRC-24-0351)

**Supplementary Figure 15:** Diagnostic tests per life-saved with increased relative hazard for cfDNA detectable cancers, CSO-directed workups.


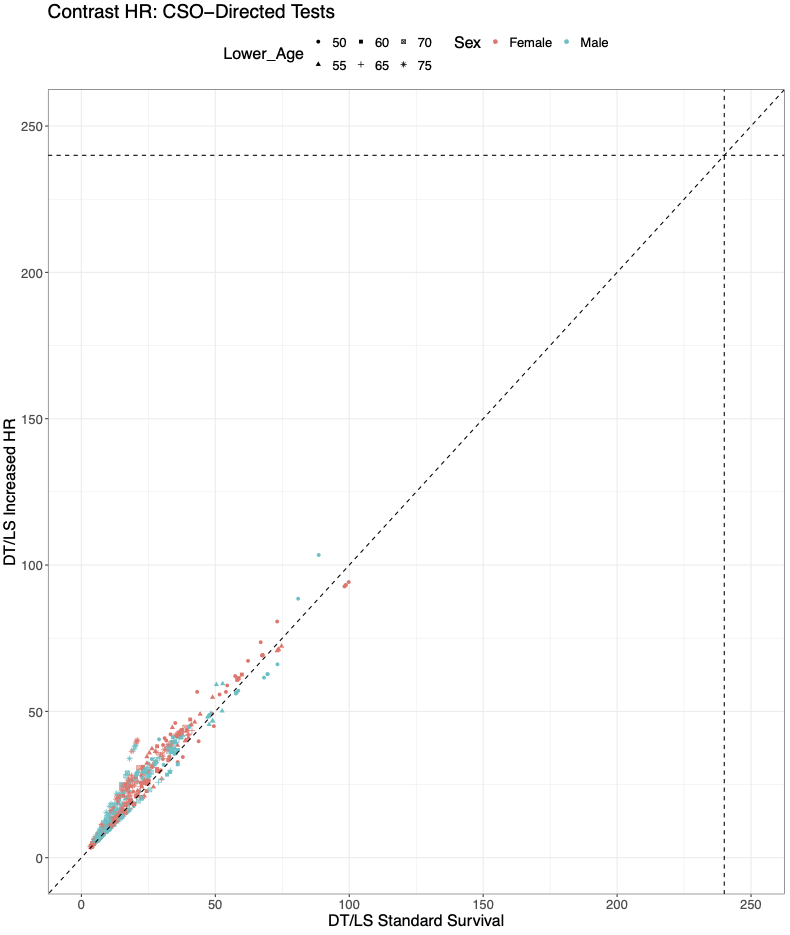

Supplement: Supplementary Figure 15 — Diagnostic tests per life-saved with increased relative hazard for cfDNA detectable cancers, CSO-directed workups [file crc-24-0351_supplementary_figure_15_suppsf15.docx]
