# Supplementary material for: Modeled Benefit of Individual Cancer Signal Origin Prediction for Multi-Cancer Early Detection
Source: Cancer Res Commun. 2025 May 19;5(5):814–24. doi: 10.1158/2767-9764.CRC-24-0351 (PMC12087281; doi:10.1158/2767-9764.CRC-24-0351)

**Supplementary Figure 16**: Diagnostic tests per life-saved with increased relative hazard for cfDNA detectable cancers, post-CSO-directed tests.


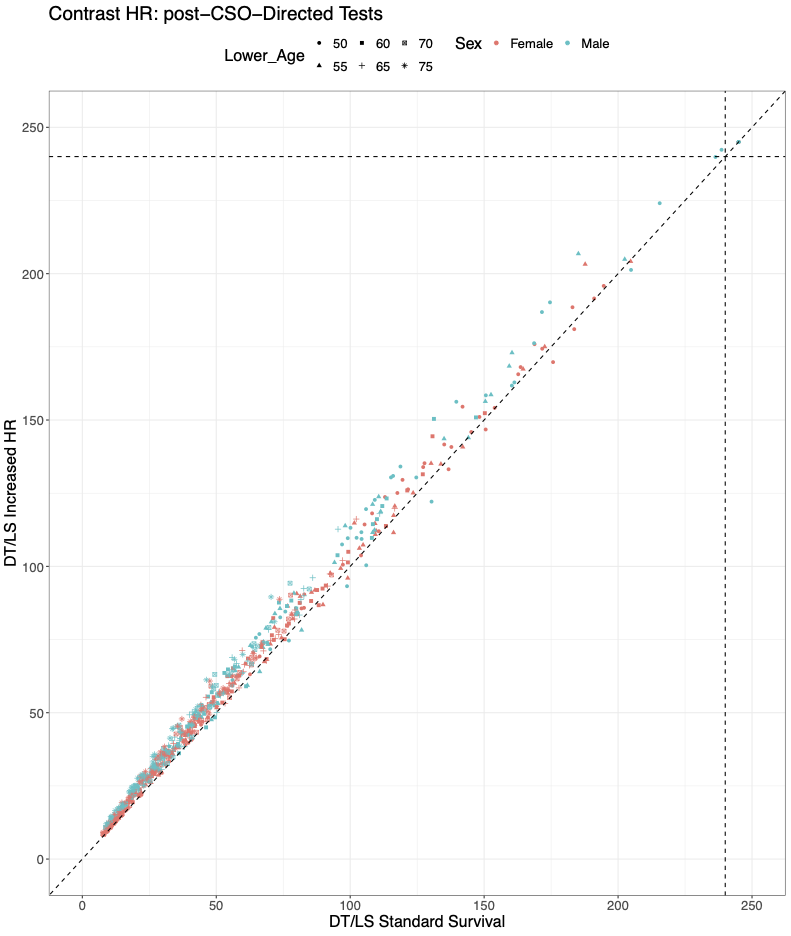

Supplement: Supplementary Figure 16 — Diagnostic tests per life-saved with increased relative hazard for cfDNA detectable cancers, post-CSO-directed tests [file crc-24-0351_supplementary_figure_16_suppsf16.docx]
