# Supplementary material for: Modeled Benefit of Individual Cancer Signal Origin Prediction for Multi-Cancer Early Detection
Source: Cancer Res Commun. 2025 May 19;5(5):814–24. doi: 10.1158/2767-9764.CRC-24-0351 (PMC12087281; doi:10.1158/2767-9764.CRC-24-0351)

**Supplementary Figure 17:** Breakdown of total numbers of tests by type under either a strategy using CSO-directed workups followed by post-CSO non-CSO-directed workups (see Methods in main paper), or a strategy using only non-CSO-directed workups. Note that CSO-directed workups are expected to be much less expensive and difficult to access than non-CSO-directed in practice, so substituting an increased number of CSO-directed workups may be favorable to reduce non-CSO-directed workups.


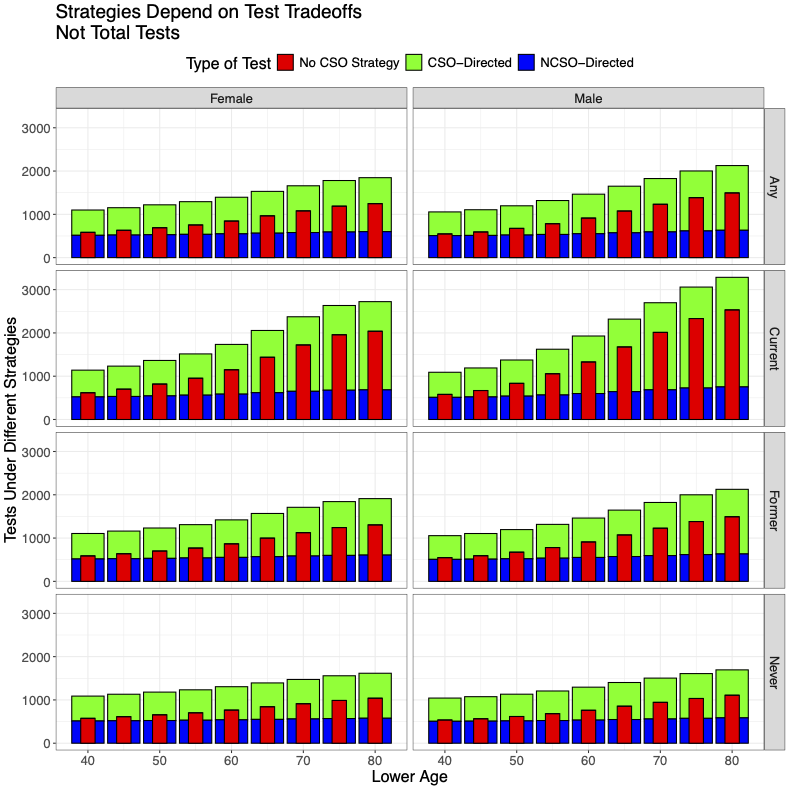

Supplement: Supplementary Figure 17 — Breakdown of total numbers of tests by type under either a strategy using CSO-directed workups followed by post-CSO non-CSO-directed workups [file crc-24-0351_supplementary_figure_17_suppsf17.docx]
