# Supplementary material for: Modeled Benefit of Individual Cancer Signal Origin Prediction for Multi-Cancer Early Detection
Source: Cancer Res Commun. 2025 May 19;5(5):814–24. doi: 10.1158/2767-9764.CRC-24-0351 (PMC12087281; doi:10.1158/2767-9764.CRC-24-0351)

**Supplementary Figure 18**: Effective ratios of “expense” to favor CSO-directed workups, stratified by age and smoking exposure. When the cancer incidence rises relative to false positives, either due to age or smoking modeled here, CSO-directed workups can be favored even at lower relative expense for non-CSO-directed workups.


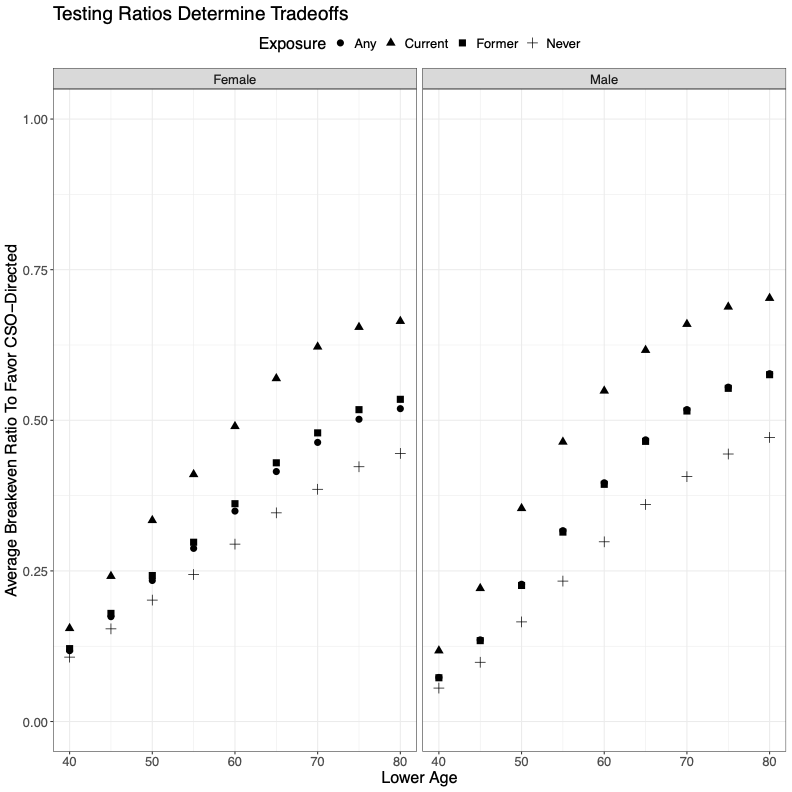

Supplement: Supplementary Figure 18 — Effective ratios of “expense” to favor CSO-directed workups, stratified by age and smoking exposure [file crc-24-0351_supplementary_figure_18_suppsf18.docx]
