# Supplementary material for: Modeled Benefit of Individual Cancer Signal Origin Prediction for Multi-Cancer Early Detection
Source: Cancer Res Commun. 2025 May 19;5(5):814–24. doi: 10.1158/2767-9764.CRC-24-0351 (PMC12087281; doi:10.1158/2767-9764.CRC-24-0351)

**Supplementary Figure 19:** Reduction in lives saved from a strategy only using CSO-directed workups and stopping any further workup, stratified by age, sex, and smoking exposure. While the majority of lives saved are retained due to the high cancer signal origin prediction accuracy, the remaining individuals with undiscovered cancer are at noticeable risk.


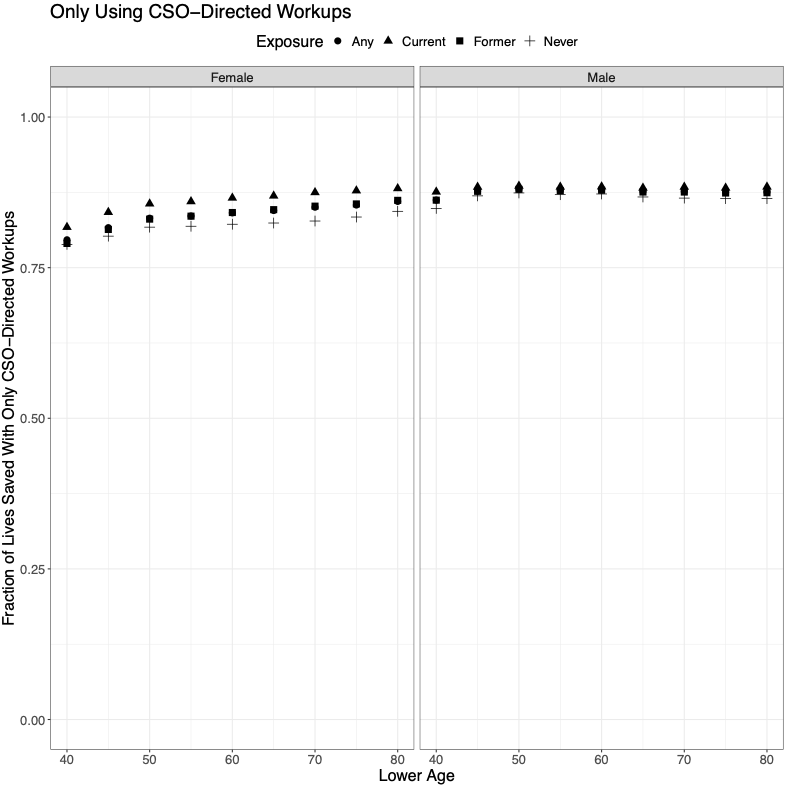

Supplement: Supplementary Figure 19 — Reduction in lives saved from a strategy only using CSO-directed workups and stopping any further workup, stratified by age, sex, and smoking exposure [file crc-24-0351_supplementary_figure_19_suppsf19.docx]
