# Supplementary material for: Modeled Benefit of Individual Cancer Signal Origin Prediction for Multi-Cancer Early Detection
Source: Cancer Res Commun. 2025 May 19;5(5):814–24. doi: 10.1158/2767-9764.CRC-24-0351 (PMC12087281; doi:10.1158/2767-9764.CRC-24-0351)

**Supplementary Figure 20:** Residual risk of cancer in individuals after CSO-directed testing, stratified by age, sex, and smoking exposure. As cancer incidence increases relative to false positive rates, the residual risk of cancer on average can be higher than usual clinically actionable levels.


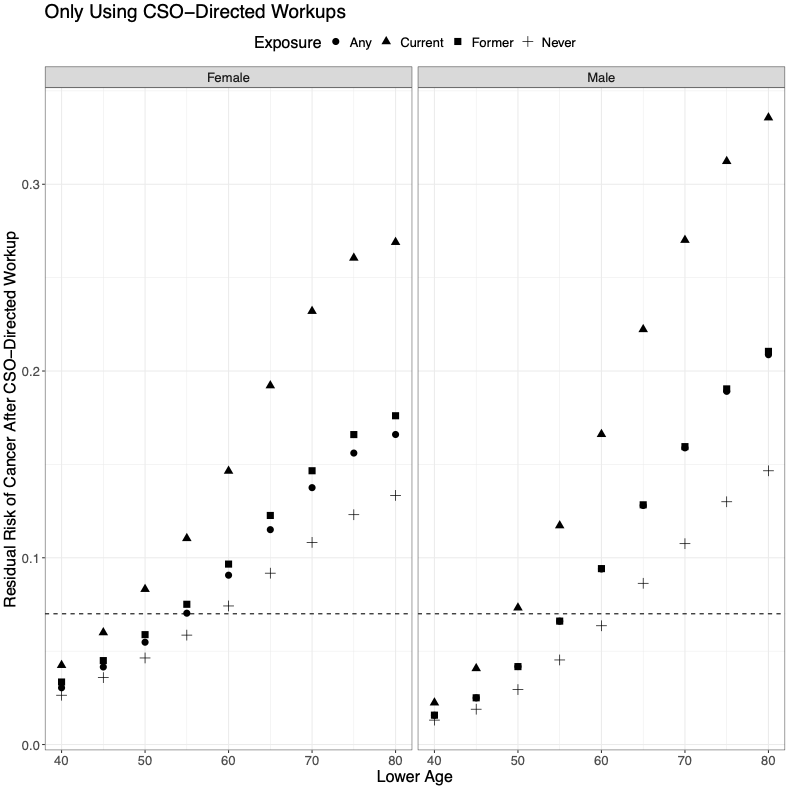

Supplement: Supplementary Figure 20 — Residual risk of cancer in individuals after CSO-directed testing, stratified by age, sex, and smoking exposure [file crc-24-0351_supplementary_figure_20_suppsf20.docx]
